# Purple: A Computational Workflow for Strategic Selection of Peptides for Viral Diagnostics Using MS-Based Targeted Proteomics

**DOI:** 10.3390/v11060536

**Published:** 2019-06-08

**Authors:** Johanna Lechner, Felix Hartkopf, Pauline Hiort, Andreas Nitsche, Marica Grossegesse, Joerg Doellinger, Bernhard Y. Renard, Thilo Muth

**Affiliations:** 1 Bioinformatics Unit (MF 1), Department for Methods Development and Research Infrastructure, Robert Koch Institute, 13353 Berlin, Germany; LechnerJ@rki.de (J.L.); HartkopfF@rki.de (F.H.); p.hiort@web.de (P.H.); MuthT@rki.de (T.M.); 2 Centre for Biological Threats and Special Pathogens, Highly Pathogenic Viruses (ZBS1), Robert Koch Institute, 13353 Berlin, Germany; NitscheA@rki.de (A.N.); GrossegesseM@rki.de (M.G.); 3 Centre for Biological Threats and Special Pathogens, Proteomics and Spectroscopy (ZBS 6), Robert Koch Institute, 13353 Berlin, Germany; DoellingerJ@rki.de (J.D.)

**Keywords:** virus proteomics, mass spectrometry, virus diagnostics, data analysis, targeted proteomics, peptide selection, parallel reaction monitoring

## Abstract

Emerging virus diseases present a global threat to public health. To detect viral pathogens in time-critical scenarios, accurate and fast diagnostic assays are required. Such assays can now be established using mass spectrometry-based targeted proteomics, by which viral proteins can be rapidly detected from complex samples down to the strain-level with high sensitivity and reproducibility. Developing such targeted assays involves tedious steps of peptide candidate selection, peptide synthesis, and assay optimization. Peptide selection requires extensive preprocessing by comparing candidate peptides against a large search space of background proteins. Here we present Purple (Picking unique relevant peptides for viral experiments), a software tool for selecting target-specific peptide candidates directly from given proteome sequence data. It comes with an intuitive graphical user interface, various parameter options and a threshold-based filtering strategy for homologous sequences. Purple enables peptide candidate selection across various taxonomic levels and filtering against backgrounds of varying complexity. Its functionality is demonstrated using data from different virus species and strains. Our software enables to build taxon-specific targeted assays and paves the way to time-efficient and robust viral diagnostics using targeted proteomics.

## 1. Introduction

Virus infections present serious health threats to millions of individuals worldwide. For public health, the accurate detection of pathogenic viruses is time-critical because reducing the time for diagnosis and treatment lowers the risk of disease transmission and patient mortality. Fast and robust diagnostic assays are therefore required to rapidly detect re-emerging and newly emerging viruses (e.g., Influenza, Ebola, Zika, or Hepatitis C virus). These diagnostic methods need to cover a broad spectrum of potentially disease-causing viral agents.

Classical diagnostic strategies for detecting viral infection can be divided into two different categories: on the one hand, virus detection can be established by targeted methods, such as agent-specific polymerase chain reaction (PCR) or immunological techniques. On the other hand, detection approaches exist that provide an open view, such as electron microscopy or next-generation sequencing (NGS). Besides their unbiased view, the latter methods have the advantage of identifying multiple pathogens in a single experimental run. Due to its specificity (hybridization and sequencing) and sensitivity (qPCR), the detection of nucleic acids is the gold standard in diagnostics. Conversely, the detection of viral proteins is used less frequently in diagnostic settings and is usually based on interaction with affine binding molecules such as antibodies or aptamers. However, producing these binding molecules is generally time-consuming and laborious, as is the validation of their specificity.

While in clinical microbiology the analysis of subproteomes (<12 kDa) using matrix assisted laser desorption/ionization-time of flight (MALDI-TOF) mass spectrometry (MS) has become a standard method for the identification of bacteria and fungi, no comparable proteomic approach exists in virology for technical reasons [1]. In recent years, MS-based targeted proteomics has evolved into a technique for detecting proteins in complex samples with high sensitivity, quantitative accuracy, and reproducibility [2,3]. Targeted proteomics is commonly used to test hypotheses on a subset of proteins of interest, in contrast to discovery shotgun proteomics. The latter provides global proteome profiling of thousands of proteins in a sample, however, at the expense of sensitivity and reproducibility. Unlike discovery methods, targeted methods of selected/multiple reaction monitoring (SRM/MRM) [4] and parallel reaction monitoring (PRM) [5] nowadays allow for detecting and analyzing preselected proteins and peptides in sensitive, specific, and time-efficient manner. Furthermore, the development of targeted proteomics assays has become easier in the past few years, owing to the advances of analytical methods, instrumental capabilities, and computational workflows [6].

Targeted MS-based proteomics assay development typically involves (i) peptide candidate selection, (ii) peptide synthesis, and (iii) assay optimization. This procedure now enables the transfer of a process highly similar to the design of multiplex PCRs to the proteome level for detecting pathogens. While MS-based targeted assays have not been used for detecting viruses in any diagnostic setting yet, promising findings could already be achieved for identifying and quantifying pathogenic bacterial species. For example, targeted proteomics methods were successfully used in previous studies on *Streptococcus pyogenes* [7] and *Mycobacterium tuberculosis* [8].

Although targeted proteomics has gained much popularity with many use cases in experimental research by now, relatively few research-oriented algorithms and software tools have been developed that support the user-defined selection of peptides for designing targeted SRM or PRM assays. In this context, Skyline [9] is a powerful and widely used software for designing targeted proteomics assays. Besides its wide applicability to different targeted methods and its intuitive use, it also has some internal limitations: first, Skyline is dependent on the operating system Windows, and can therefore not be used under a Linux cluster server environment, and second, it does perform only exact string matching during the peptide selection process without considering any homologies between related organisms. PeptidePicker [10] is a web-based workflow to select peptides by providing, amongst further options, the protein accession number and was designed for human and mouse proteomes. PeptideManager [11] is a tool developed to select peptide candidates as protein surrogates from a defined proteome. It was optimized for the use case of xenografts, i.e., human tumors orthotopically implanted into a different species. While this software allows for constructing a peptide database from any species-specific proteome, sequence homologies, and multiple taxonomic levels are disregarded. Picky [12]—a web-based method designer for targeted assays—only provides support for human and mouse sequences, while it relies on synthetic peptide data from the human-focused ProteomeTools project [13,14]. In the context of targeted metaproteomics, the Unique Peptide Finder of the UniPept web application [15] was developed to select unique peptides for user-defined taxa. Furthermore, various computational tools have been developed to predict proteotypic peptides for targeted proteomics experiments [16,17,18]. These methods often make use of machine learning training setups that incorporate the probability of observing a peptide in a standard proteomics analysis, referred to as peptide detectability [19] or observability [20], and commonly involve physicochemical properties of the proteins to select high-responding peptides [21]. To our best knowledge, however, no software tool is currently available to select taxon-specific peptides for targeted proteomics assays that also accounts for sequence homologies between different species or strain proteomes. Effectively considering homologies is crucial for accurate taxon-specific diagnostics, because proteins measured in virus samples frequently have a high sequence similarity either in closely related strains or due to highly conserved functional domains. 

Here we present Purple (Picking unique relevant peptides for viral experiments), a platform-independent software that returns a set of taxon-specific peptides, after the user has specified the viral target (i.e., a particular virus species or genus), as candidates for targeted proteomics experiments. Equipped with a user-friendly graphical user interface and a threshold-based filtering strategy for homologous sequences, it simplifies the design of MS-based targeted proteomics assays for the end user. Purple enables peptide candidate selection and considers background sequence information, i.e., proteins that are not related to a specific virus target, at various taxonomic levels. Thus, all peptide candidates are validated against a user-defined database of virus proteomes. While the design of MS-based targeted assays requires further steps, our software greatly facilitates the cumbersome, yet important task of peptide selection and thereby paves the way to time-efficient and robust pathogen screening and viral diagnostics. Purple is open source software available at https://gitlab.com/rki_bioinformatics/Purple.

## 2. Materials and Methods

### 2.1. Purple Workflow

**Figure 1:**
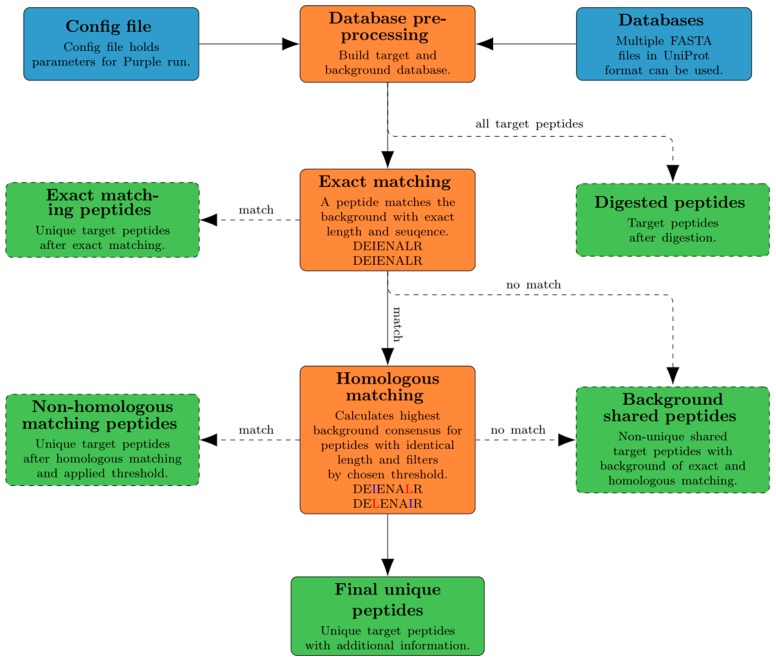
Overview of the Purple workflow. A configuration file and a directory path to the location of FASTA databases serve as input (blue). In the database preprocessing step, the databases are separated into target and background (orange). Any target peptides exactly matching to the background database are removed. In the homologous matching step, any target peptides that have similar sequences are filtered out (orange). All intermediate and final results are exported automatically to a user-defined output folder (green).

Purple is implemented in Python (version 3.6) and makes use of additional Python libraries such as tqdm (https://github.com/tqdm/tqdm) for process bar calculation and Biopython [22] to calculate the molecular weight of peptides. Purple is available as portable standalone version that already includes all required libraries or Purple can be installed using pip or conda, which are managing dependencies. The workflow of Purple is depicted in an overview diagram (Figure 1). Purple requires the input of protein sequence databases and a configuration file. The databases are automatically rearranged into a target and a background database. The “exact matching” step is used to remove exact sequence matches with the background from the target peptide set. The remaining target peptides are used to detect and remove homologous peptides. A result file containing the final unique peptides is created together with various intermediate result files. These are outputs of all Purple processing tasks, namely (i) digested peptides, (ii) exact matching peptides, (iii) non-homologous matching peptides and (iv) background shared peptides.

#### 2.1.1. Preprocessing (Target Selection)

The selection of a target virus proteome is handled by input and preprocessing routines in Purple. For target selection, protein sequence databases in FASTA format serve as main input and are required to be provided in UniProt format. To select the database input, a directory needs to be specified by the user and multiple FASTA files can be considered for the processing. Two options of database specification are available in Purple: the first option is to explicitly define target species names as a list, which leads to the merging of all provided input databases. Each protein entry that contains one of the defined target species names in the protein header is considered as a target protein. The protein sequences not matching the defined target species are used as background database. The second option is to select a specific FASTA file in the database directory as target database. All remaining databases in the directory are then automatically merged to a single background database. As the background database may still consist of proteins originating from one of the target species, each protein in the background database is checked once more: if a protein header matches any species in the specified target database file, the protein entry is removed from further processing accordingly.

Both options result in two types of databases, namely a target and a background database. In the following, each protein sequence in these databases is *in silico*-digested using the enzymatic rule of trypsin with optional proline digestion. The in silico digest step results in multiple peptides for each protein entry, and peptide sequences beyond the user-defined length boundaries are filtered out. In addition, preprocessing includes the option of removing protein fragments and also allows replacing each isoleucine by leucine: this option was implemented because these amino acids share identical molecular masses and are therefore commonly not distinguishable in mass spectrometry. When the preprocessing is completed, both a target and a background database are provided for further analysis, which in this stage consist of peptides instead of proteins.

#### 2.1.2. Exact Matching

Exact matching presents the first actual processing step in Purple: here, each of the previously *in silico*-digested target peptides is compared against the provided background database (see previous paragraph). In this procedure, target and background peptides of identical length are compared and only those target peptides that are not contained in the background are considered further; thus, peptide sequences with one or more exact sequence matches in the background database are filtered out at this stage, because they are not unique to the user-defined taxa of the target space. This procedure is performed iteratively until all *in silico*-digested peptides have been evaluated. The remaining peptides that have not been filtered out are stored as unique peptide candidates for further processing and are exported as intermediate result of the exact matching step.

#### 2.1.3. Homologous Matching

Homologous matching is performed subsequently to the exact matching step. The goal is to evaluate each of the unique peptide candidates concerning its potential sequence consensus to homologous proteins in the background. The rationale behind this approach is that the more similar a target peptide is to the background, the less appropriate it is as candidate for a taxon-specific targeted assay. To assess the similarity of each peptide to the background proteomes, a sequence background consensus metric is introduced (see next paragraph). The target peptides that are discarded either during the exact or the homologous matching step are exported as so-called “shared” peptides. Shared peptides have either an exact sequence match with the background or have background consensus value above a user-defined threshold. To keep track of all processed data, target peptides with a background consensus below the threshold are exported as well.

#### 2.1.4. Background Consensus Metric and Threshold Generation

Owing to mutational effects on conserved viral proteins, peptides can often be shared within a virus genus or family with minor sequence variations between them. This is problematic for targeted assays because such peptide candidates are not specific for species- or strain-level identification. To remove such taxon-unspecific peptides from the final sequence set, the background consensus metric f(A,B) is used in Purple as the essential part of the homologous matching. Basically, the background consensus presents the Hamming distance of a target peptide A and background peptide B of the same length in relation to the length of the peptide n (Equation (2)). An amino acid is shared if the same amino acid (d(x,y)) is at the same position in A and B (Equation (1)).

(1) $$d\left(x,y\right)=\{\begin{array}{ll} 1, & if\;x=y\\ 0, & if\;x\neq y \end{array}$$

In other words, the background consensus is the sum of shared amino acids at a specific position i divided by the number of amino acids in both (target and background) peptides. Even though the Hamming distance is a simple metric, it provides a proof-of-concept and validation of Purple, as adding more sophisticated methods should only slightly improve the homologous matching while increasing the computational effort and complexity.

(2) $$For\;A=\left\{a_{1},a_{2},\ldots ,a_{n}\right\}\;and\;B=\;A=\left\{b_{1},b_{2},\ldots ,b_{n}\right\}\;and\;n=\left|A\right|=\left|B\right|:\;f\left(A,B\right)=\frac{\sum_{i=1}^{n}d\left(a_{i},b_{i}\right)}{n},for\;a_{i}\in A\;and\;b_{i}\in B$$

This metric is applied to each of the target peptides that are compared to all background peptides of the same length. For each target peptide, the maximum consensus is stored when being below a user-defined background consensus threshold. A target peptide with a high background consensus is likely to originate from a homologous protein or common protein domain. Therefore, the consensus metric evaluates the conservation of peptides in the target and background database. A low background consensus marks target peptides that are unique in sequence in the target species. All peptides with a high background consensus below the previously chosen threshold are exported into the final results file and the remaining shared peptides are exported as part of the intermediate output. The results are supplemented with the peptide weight, the number of occurrences in the target database, as well as species and proteins names. This enables the user to conduct further analysis with the previously retrieved unique peptides. The Purple documentation is available for a complete description of all output files and more details about the data interpretation.

### 2.2. Graphical User Interface

**Figure 2:**
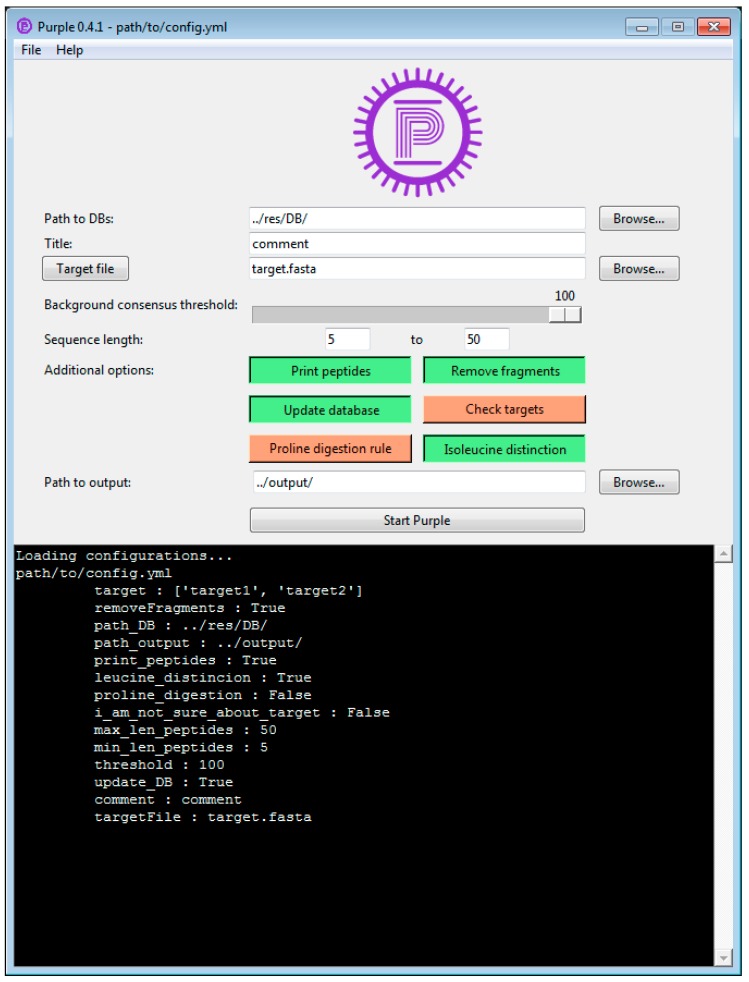
The graphical user interface of Purple. In the top file menu, configurations files can be loaded and saved. The top menu also includes a link to the documentation and manual. The listed GitLab page provides direct user support from the developers via an issue tracking system. The upper panel shows default parameters and allows modifying the configuration settings and processing start. The lower panel displays the current processing status with logging information on the current run, configuration, and progress of the analysis.

**Figure 3:**
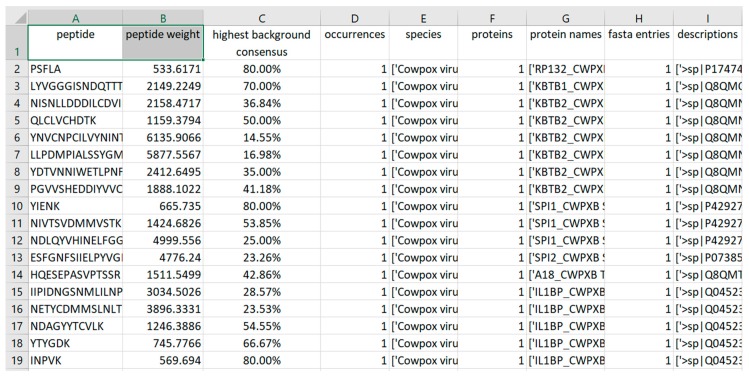
Graphical representation of the Purple output. The tabular TSV output of Purple can be imported into various spreadsheet software tools. This exemplary table shows the peptide sequence, the calculated theoretical mass weight (Da), the highest background consensus, and the number of peptide occurrences in the target proteome. The species, protein name and full description of the associated protein are stored in a list for further analysis. In addition, the number of proteins and FASTA entries are listed separately, because they can diverge, e.g., when a protein has multiple sequence variants.

A graphical user interface (GUI) was developed for using Purple (Figure 2). This interface allows researchers with less expertise in handling bioinformatics methods on the command line to use Purple in a efficient and user-friendly manner. The Purple GUI makes software configuration and execution straightforward and complex tasks can be rapidly accomplished. Any parameter can be adjusted in the GUI, and the background consensus threshold can be set by the user. Furthermore, the processing status can be inspected in a logging panel and a file menu provides options for saving and loading configuration files. Note that configuration files are optional in Purple and a default configuration is provided; thus, only system-specific parameters must be set in the GUI. Using configuration files makes each task reproducible and the GUI-integrated configuration file choice allows for switching between multiple settings easily. Figure 3 shows the final output in the tab separated values (TSV) format that can be further processed and visualized using common spreadsheet software.

### 2.3. Data

#### 2.3.1. Target Virus Databases

To evaluate the performance of Purple, selected target virus species from sequence databases were used. This section provides an overview on the virus species used with respect to database composition and further background information on the virus type. The virus species were selected based on their relevance for current or upcoming diagnostic settings.

##### Arenaviruses

Arenaviruses are enveloped RNA viruses with an average diameter of 120 nanometers that have a bisegmented negative-strand RNA genome. The Latin term “arena” refers to the grainy ribosomal particles acquired from the virus-host cells that can be viewed in cross-section with electron microscopy imaging. Arenaviridae is a virus family whose members are generally associated with causing chronic infections in rodents and zoonotically acquired severe diseases, such as lymphocytic choriomeningitis or hemorrhagic fever, in humans. In this work, nine disease-causing Old and New World arenavirus species are taken as targets for evaluating the performance of Purple (Table 1). Besides Lymphocytic choriomeningitis virus, strain members of which cause aseptic meningitis, encephalitis, or meningoencephalitis, all listed arenaviruses are causative agents for viral hemorrhagic fever (VHF).

##### Cowpox virus

Cowpox virus (CPXV) is a large double-stranded DNA virus with a proteome of over 200 proteins [24] that belongs to the genus Orthopoxvirus (OPV) of the Poxviridae family. CPXV has been described as the source of the first vaccine used by Edward Jenner, who was the first to scientifically describe the vaccination process against the smallpox-causing variola virus. Recent findings based on a conducted analysis on the smallpox vaccine gave evidence of the suspected role of horsepox (instead of cowpox) in the origin of the vaccine [25,26]. Since the pathogenicity and zoonotic potential of CPXV are investigated at the Robert Koch Institute, detailed data acquired from MS measurements were available (see Section 2.3.3). For performance evaluations, CPXV is further beneficial because this virus species has several close relatives. In addition to the cowpox strains Brighton Red and Grishak-90, four very close relatives with high sequence similarity are given: a genome comparison performed with BLAST [27] showed that variola virus, monkeypox virus, horsepox virus, and vaccinia virus share sequence identities of up to 98% (Supplementary Table S1). 

##### Vaccinia virus (VACV Copenhagen and VACV Western Reserve)

Vaccinia virus is a member of the Orthopoxvirus (OPV) genus [28] and has been used for vaccination against smallpox since the 19th century. Due to the high sequence similarity of members of the OPV genus, it is possible to provide cross-protection vaccination by one member of the OPV genus. Hence, the classification can be an issue, because it can be challenging to find peptides to reliably classify a species or a strain. In this work, we investigate whether it is possible to distinguish between the two strains VACV Copenhagen and VACV Western Reserve by finding strain-specific peptides using Purple. Similar to CPXV, experimental data was publically available (see Section 2.3.3).

#### 2.3.2. Background Virus Databases

The target databases mentioned above are species-specific and therefore cannot represent all available virus proteomes. From the target databases, Purple only yields to species-specific unique peptides. To extend this space to all virus proteomes and subsequently be able to find unique peptides in that relation, we added a database that consists of all reviewed virus proteins available on UniProt/Swiss-Prot [29]. In contrast to the target databases, this database is used exclusively as a background database. At the time of writing, UniProt/Swiss-Prot contains 16,846 reviewed viral proteins, which results in 301,387 in silico-digested tryptic peptides. In this work, we evaluate Purple with and without the use of the larger background database. 

#### 2.3.3. Background Human Databases

To account for samples mixed with human proteins we added a human database to the background. This database originates from UniProt/Swiss-Prot [29] and enables Purple to discard human peptides. Subsequently, this reduces false positives in experiments using virus-infected human samples. The database consists of 20,428 proteins and was used exclusively for the CPXV analysis in this work.

#### 2.3.4. Experimental Data

The MS/MS datasets used for the benchmarking of Purple originate from a previous study published by Doellinger et al. in 2015 [24] (PRIDE project accession: PXD003013). In this work, a subset of the data available was used including three CPXV Brighton Red, three VACV Copenhagen, and three VACV Western Reserve MS/MS raw files. These raw files were acquired by an LTQ Orbitrap in data-dependent manner. Further experimental details are listed and described in the above-mentioned publication. Subsequently, three CPXV Brighton Red raw files were converted into MGF files using the MSConvert function of ProteoWizard [30] with the peak picking parameter of MS-level two and with zero sampling removal activated. Table 2 shows the number of MS/MS spectra for each virus strain (CPXV Brighton Red, VACV Copenhagen and VACV Western Reserve). For peptide and protein identification, these spectra were searched against proteome databases using the MS-GF+ [31] (version v20181015) database search engine. The database search was performed with eight threads, an activated decoy search, a chosen precursor with mass tolerance of five ppm, optimized for Orbitrap instruments, and trypsin was selected as digestion enzyme. The sequence databases used for protein identification are described in detail in Section 2.3.1. The database searches produced mzid output files that were converted into TSV files using the build-in MS-GF+ conversion tool. Afterwards, the results were filtered by applying a 1% false discovery rate (FDR) threshold at the PSM-level.

## 3. Results

We here present three different use cases to illustrate the possibilities of targeted proteomics using Purple in viral diagnostic settings. The first analysis focuses on the species-level resolution for arenaviruses, the second evaluates the taxonomic classification using cowpox data from shotgun proteomics measurements, and the third tests the capabilities of strain-level differentiation using experimental data from two closely related vaccinia virus strains. 

### 3.1. Analysis of Species-Level Resolution using Nine Arenavirus Species

In the first analysis, we aimed to evaluate the species-level resolution of our diagnostic approach using sequence data from the Arenaviridae family. For this purpose, we investigated the resolution of Purple by evaluating different viral species as target organisms against a proteome background of similar species and viruses in general. We used nine arenavirus species (MACV, JUNV, SABV, CHAV, GTOV, LASV, LCMV, WWAV, and LUJV; see Table 1) with proteomes containing four proteins, namely (1) RNA-directed RNA polymerase L, (2) nucleoprotein N, (3) pre-glycoprotein polyprotein GP complex and (4) RING finger protein Z. As background proteomes, we added all reviewed virus proteins available on UniProt/Swiss-Prot to remove frequently occurring peptides (e.g., from conserved sequences of functional domains). The removal of target peptides from similar virus proteomes intends to eliminate false positive detections (i.e., to increase the specificity). Since the protein sequences differ strongly between the arenavirus species, we expected to retrieve sufficient unique peptides for each species that serve as candidates for designing a targeted assay. For a benchmarking, we examined the relative amount of taxon-specific target peptides for each of the arenavirus species using both exact and homologous matching mode (Table 3 and Table 4). The homologous matching was performed to evaluate the impact of sequence homologies for the arenaviruses and between these and all other virus species.

**Figure 4:**
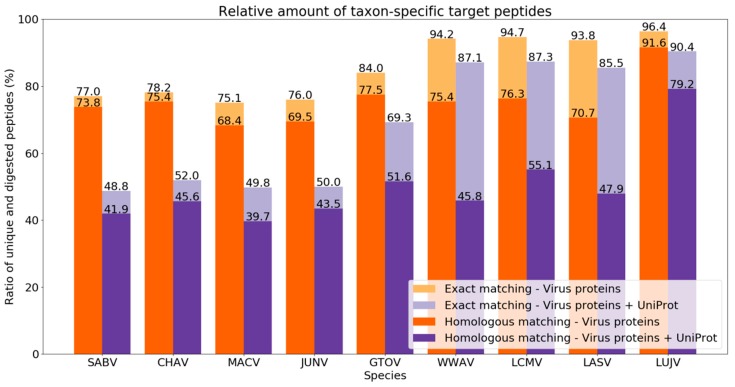
Relative amount of taxon-specific target peptides from nine arenavirus species proteomes. The ratio of unique to in silico-digested peptides is shown for exact (lighter colors) and homologous (darker colors) matching mode with a background consensus threshold of 80%. Orange bars show the results for the database consisting of four virus proteins for each arenavirus species. Purple bars indicate results that were generated when adding protein sequences from all reviewed virus proteomes (from UniProt/Swiss-Prot) as additional background.

**Figure 5:**
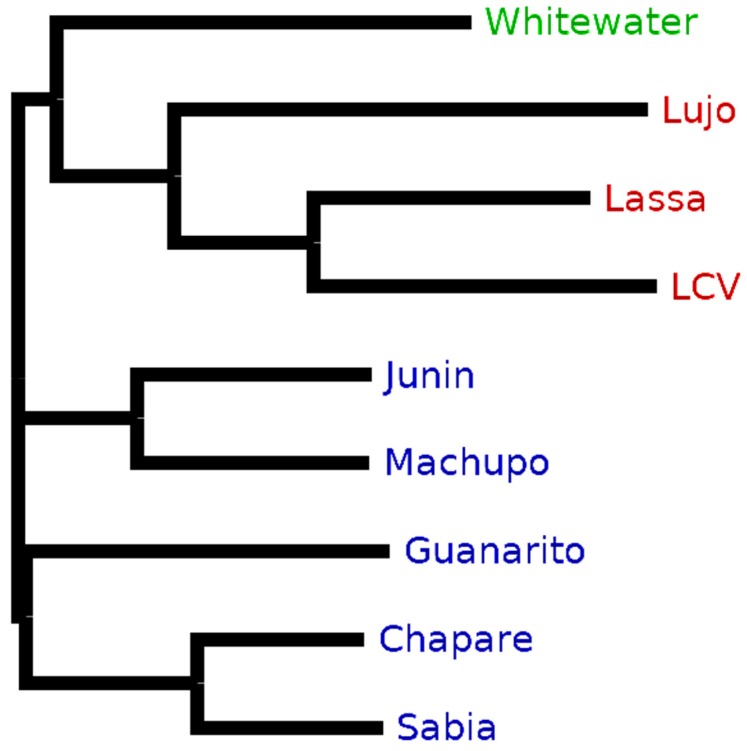
Phylogenetic tree of the pre-glycoprotein polyprotein GP complex (GLYC) of nine arenaviruses. The Whitewater strain is the only New World clade A/rec arenavirus (green). Lujo (LUJV), Lassa (LASV), and Lymphocytic choriomeningitis (LCV) are geographical Old World arenaviruses (red). Junin (JUNV), Machupo (MACV), Guanarito (GTOV), Chapare (CHAV), and Sabia (SABV) are members of the New World arenaviruses clade B (blue). The neighbor-joining tree without distance corrections was created using CLUSTAL Omega [34] for the multiple sequence alignment and the tree visualization software FigTree (http://tree.bio.ed.ac.uk/software/figtree/).

First, we investigated the ratios of taxon-specific unique peptides and in silico-digested peptides with a background database consisting of the four arenavirus proteins, as mentioned above. The exact matching yielded to taxon-specific peptide ratios between 75.1% (MACV) and 96.4% (LUJV) (Figure 4). This can be explained by the high sequence diversity between the nine arenavirus species: when generating multiple sequence alignments (MSA) of these species for their four proteins, overall, a low consensus of the sequences was found (Supplementary Data S1–S4). When applying a background consensus threshold of 80%, significantly fewer taxon-specific peptides were obtained with relative numbers between 48.8% and 90.4% for SABV and LUJV, respectively (Figure 4). Overall, the mean decrease in the ratio of all species is 16.6% and the strongest ratio decrease can be found for MACV (25.3%), SABV (28.2%), CHAV (26.2%), and JUNV (26.0%). These four species are all New World arenaviruses and part of the clade B (see Table 2). The close relationship of these four virus species (as shown in the phylogenetic tree in Figure 5) causes high numbers of shared peptides which explains the decline in taxon-specific peptides. The Old World arenavirus LUJV shows the highest taxon-specific peptide ratio after homologous matching (90.4%) and even after homologous analysis against all virus proteomes (79.2%). This illustrates that LUJV has the lowest sequence similarity with the other arenaviruses. The low similarity can be explained by the isolated geographical distribution of LUJV in Southern Africa [32]. In 2008, an outbreak of LUJV led to a high case fatality rate of 80% (4/5 cases), and a follow-up analysis of its genome confirmed that LUJV is a novel virus species being only distantly related to known arenaviruses and groups genetically closer to Old World viruses not associated with VHF [33].

Next, we assessed the protein sequence coverage on the basis of Purple-selected unique peptides for all four arenavirus proteins (RNA-directed RNA polymerase L; Nucleoprotein N; Pre-glycoprotein polyprotein GP complex GLYC; RING finger protein Z). We evaluated two different backgrounds here: (i) a small background with the arenavirus proteomes (containing the four proteins) of the remaining eight non-target species and (ii) a large background containing all arenavirus proteomes combined with all reviewed virus proteomes from UniProt/Swiss-Prot (see Section 2.3.2).

**Figure 6:**
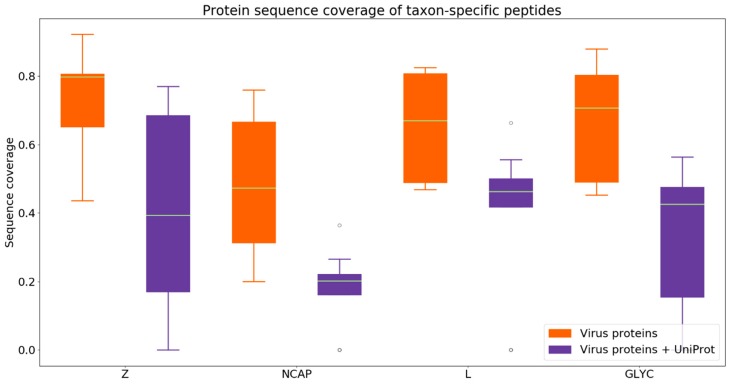
Protein sequence coverage of taxon-specific peptides selected by Purple on proteins for nine arenavirus species proteomes. The four proteins of the arenavirus proteomes are RNA-directed RNA polymerase L (L), nucleoprotein N (NCAP), pre-glycoprotein polyprotein GP complex (GLYC), and RING finger protein Z (Z). The coverage of selected peptides is displayed for homologous matching when applying a background consensus threshold of 80%.

The analysis of the protein sequence coverage shows that L, GLYC and Z are relatively well covered by the taxon-specific peptides across all nine species for the small background (Figure 6). Nucleoprotein NCAP has the highest variability in protein coverage with an interquartile range (IQR) of 35.22% on the small background, suggesting that NCAP is the best-conserved protein among the considered arenavirus species. When taking a closer look at the results of the larger background analysis with all reviewed virus proteins, it can be found that the coverage decreases for all four proteins. The NCAP protein shows the lowest median in protein coverage (20.18%). This shows that NCAP has the lowest sequence consensus of taxon-specific peptides with other virus proteomes, indicating that it is the best-conserved of the four proteins. Indeed, the other three proteins (L, GLYC, and Z) have above 40% sequence coverage, thus more taxon-specific peptides can be obtained from these proteins. This analysis shows that, depending on the use case, it may make sense to investigate individual proteins instead of whole proteomes. For example, proteins with low sequence coverage based on taxon-specific peptides may be excluded 

### 3.2. Evaluating Species-Level Classification Based on Detected Peptides from Viral Shotgun Proteomics Measurements

To evaluate the peptide selection method in Purple on experimental data, we used representative MS/MS datasets derived from human cowpox virus (CPXV) samples. The main goal was to test whether peptides identified in a typical shotgun proteomics experiment can be used for differentiating viruses at the species level. We also aimed for estimating the expected accuracy gain for taxonomic classification when using a targeted proteomics assay on the basis of peptides suggested by Purple.

In a pre-analysis, we performed a Purple run using CPXV as target proteome to select species-specific peptides. For the peptide selection process, 18 reviewed (from UniProt/Swiss-Prot) and 208 unreviewed (from UniProt/TrEMBL) CPXV-specific protein sequences were used as target database, which is part of the PRIDE project (see Section 2.3.4). We used this combined database consisting of reviewed and unreviewed protein sequences because the available reviewed protein sequences for the Brighton Red strain yielded to a very limited number of peptide identifications during the database search (Supplementary Table S2). All available virus proteomes (a total of 16,846 sequences) and all reviewed human proteins were taken as background. These proteomes were obtained from UniProt/Swiss-Prot (see Section 2.3 for database details). 

The Purple run resulted in 1509 in silico-digested peptides after exact matching and 885 peptides after homologous matching (using a background consensus threshold of 80%). The distribution of the homologous background consensus shows a normal distribution below 50% (Supplementary Figure S2). 3986 peptides were discarded, because they were shared with other (i.e., non-CPXV) viral proteomes or the human proteome. The remaining 885 CPXV-specific peptides have a mean background consensus of 53.9%, which means that on average around half of the amino acids of each peptide are equal to residues of peptides in the background.

Next, we searched experimental MS/MS spectra from CPXV samples using the search algorithm MS-GF+ [31] against a CPXV and human sequence database for peptide identification (see Section 2.3). In this analysis, CPXV datasets from MS measurements of three technical replicates, each with ~19,000 MS/MS spectra, were evaluated. The database search resulted in 4028, 4125, and 3967 identified peptides per sample replicate with sequence duplicates removed. More than twice the amount of CPXV peptides were identified as human peptides in this sample before applying a FDR filtering. After applying an FDR threshold of 1%, 1067, 1028, and 1004 CPXV peptides were identified (Table 5). Subsequently, the identified peptides (below 1% FDR threshold) were compared against the set of taxon-specific CPXV peptides suggested by Purple using both exact and homologous matching mode. Between 83 and 94 peptides selected by Purple were detected in the MS/MS experiments (without applying any FDR threshold). When filtered by 1% FDR, the peptides decreased to numbers between 78 and 84. Consequently, this analysis demonstrates that it would be possible to reliably identify CPXV for these three sample replicates.

**Figure 7:**
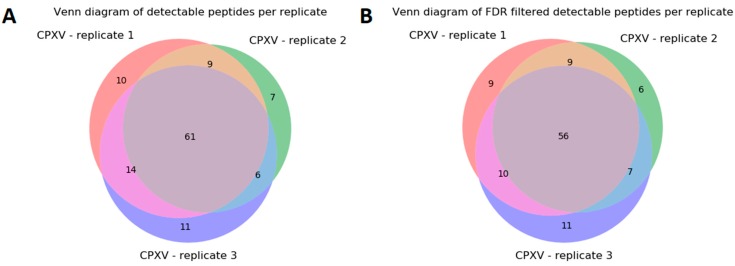
Intersection of detectable peptides of CPXV sample replicates. These Venn diagrams show the intersection of the detectable peptides in replicates 1–3. The subfigures depict the number of peptides without applying any false discovery rate (FDR) threshold (**A**) and filtered by 1% FDR (**B**).

When considering the results of all three replicates, it can be observed that 61 CPXV-specific peptides were detected without any applied FDR threshold (Figure 7A). Filtered by 1% FDR, 56 peptides across all replicates can be used to specifically identify the species within the sample as a member of CPXV (Figure 7B).

**Figure 8:**
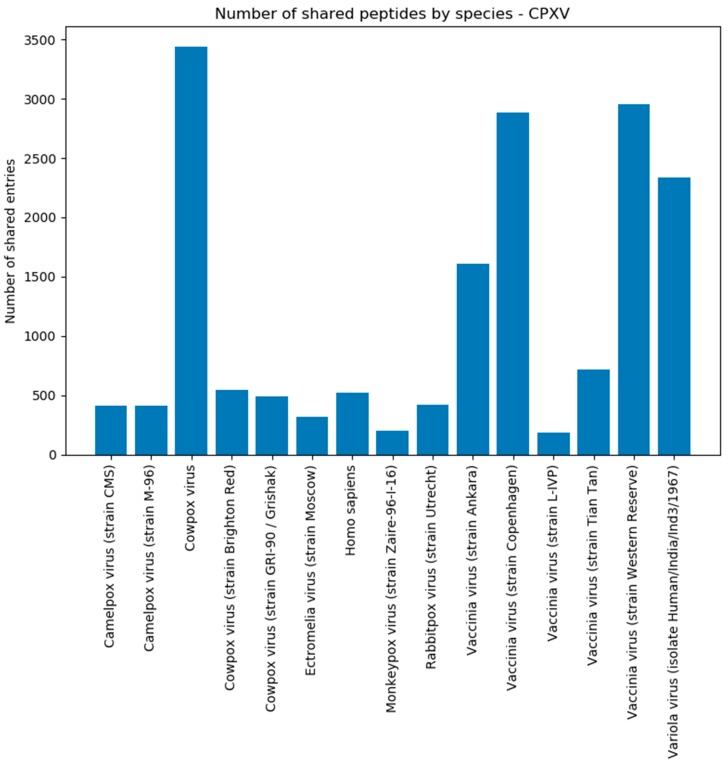
Number of shared CPXV peptides by species/strain assigned. This plot shows the number of shared peptides that Purple detected in the background for a species/species after the CPXV Brighton Red target analysis. All species that contribute less than 0.5% to the total amount of shared peptides were removed.

When examining the peptides shared by the target and background proteomes, it can be found that the Cowpox virus shares ~3000 peptide sequences per strain with the Vaccinia virus strains and Variola virus strains (Figure 8). Other Orthopoxviruses were found as well, although the number of peptides is low, due to fewer proteins of these strains in the background database. The CPXV Brighton Red strain-specific peptides are small in number because most matches originate from the Cowpox virus species proteome without giving any details about a particular strain. Around 500 peptides were shared with the human proteome and were consequently discarded.

### 3.3. Comparison of Strain vs. Strain and Strain vs. All Virus Level Resolution

Next, we conducted a performance evaluation using two different, yet highly similar Vaccinia virus strains, namely VACV Copenhagen and VACV Western Reserve. The objective was to test whether Purple can retrieve strain-specific peptides that are then used in the targeted proteomics assay for accurate taxonomic classification. In this analysis, the target database contained sequences from one of the two VACV virus strains (either Copenhagen or Western reserve). Consequently, the background database contained the remaining VACV strain and all reviewed virus proteins available on UniProt. This procedure was repeated with the remaining VACV strains as target. The goal was to find strain-specific peptides to accurately detect the virus strain. We used a background consensus threshold of 80% to filter out homologous peptides. Afterwards, experimental data (see Section 2.3.3) was used to validate the results and to show if the selected strain-specific peptides are found in the acquired tandem mass spectrometry (MS/MS) data. For peptide identification, we used the software MS-GF+ [31] with an 1% FDR threshold (see Section 2.3.3).

In the case of VACV Copenhagen, Purple discarded 3848 peptides because a perfect sequence match was present in the background with a peptide of another strain or virus (Table 6). Equally, 3971 VACV Western Reserve peptides are marked as shared with the background and discarded. After exact matching, 498 and 341 strain-specific peptides could be obtained for VACV Copenhagen and VACV Western Reserve, respectively. The homologous matching removed additional 157 (VACV Copenhagen) and 172 (VACV Western Reserve) peptides from the set of unique peptides. The remaining 352 (VACV Copenhagen) and 169 (VACV Western Reserve) peptides can be used to uniquely identify the strain in a mixture of all reviewed virus proteins available on UniProt/Swiss-Prot.

**Figure 9:**
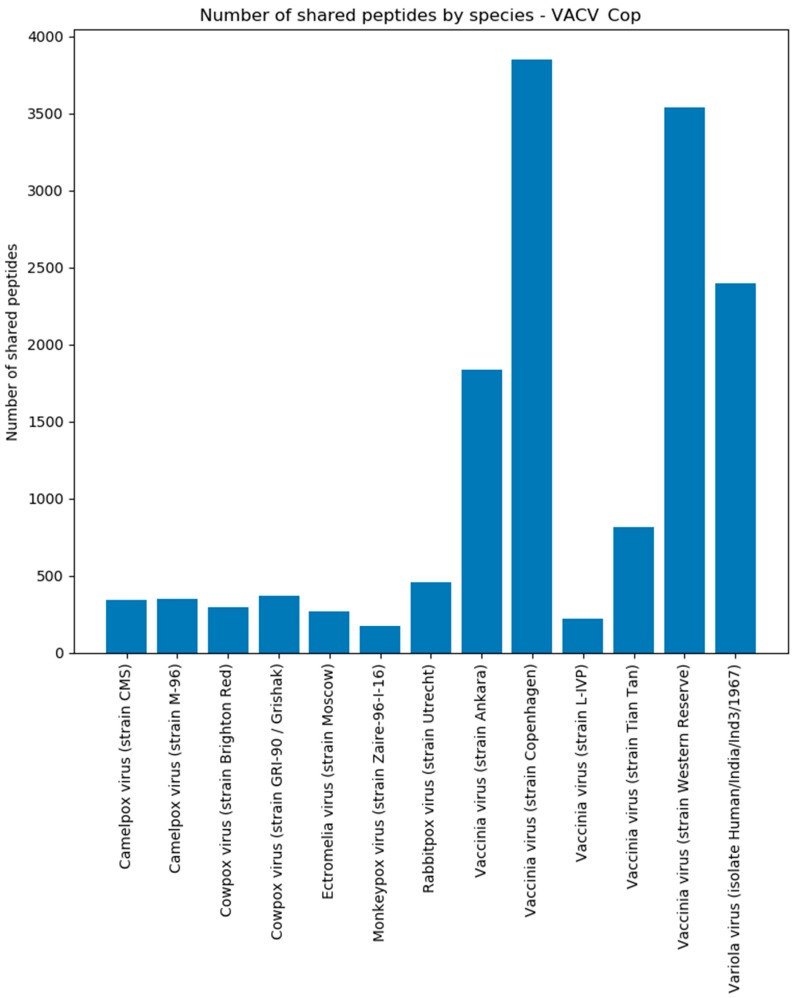
Number of shared peptides by species. This plot shows the number of shared peptides that Purple detected in the background for a species after the VACV Copenhagen analysis. All species that contribute less than 0.5% to the total amount of shared peptides were removed here.

In addition, we categorized the shared peptides by virus species to check for close relationships in the background. For VACV Copenhagen, it can be observed that most peptide matches are found in the Vaccinia species (Figure 9), owing to a high protein sequence similarity of involved Vaccinia strains. Other contributing species are Camelpox virus, Cowpox virus, Monkeypox virus, Rabbitpox virus, and Ectromelia virus. All these viruses are, as expected, members of the orthopoxvirus genus. Similar findings could be observed for the results of the VACV Western Reserve strain (Supplementary Figure S1). Note here that Figure 9 shows the number of peptides and if a species is underrepresented in the databases, it will affect the outcome concerning the number of peptides that contribute to the shared peptides. 

To evaluate the detectability of taxon-specific peptides for the given DDA experiments, we performed database searches for peptide identification using three different technical replicates of VACV Copenhagen. Without any FDR cut-off, we could identify between 60 and 66 strain-specific peptides selected by Purple (Table 7). However, when filtered by an FDR of 1% the number of peptides decreased drastically and only one or two taxon-specific peptides were confirmed in the shotgun proteomics data. It was possible to identify Replicate 1 and 2 as VACV Copenhagen by using the peptide sequence ILFWPYIEDELR. The number of peptides can be increased by switching to a targeted proteomics approach and by considering PTMs or by an improved homologous matching. The three technical replicates of the VACV Western Reserve strain resulted in fewer peptides in the intersection with the database search results (between 32 and 42), but when filtered by 1% FDR, the number of peptides was increased up to 11-fold (with nine to 11 peptides) in comparison to the VACV Copenhagen replicates. Six peptides were detected, and their sequences were identical among all three replicates.

In conclusion, we were able to identify every strain in each sample with an applied FDR of 1%. For VACV Western Reserve, the number of peptides was higher than for the VACV Copenhagen strain. The number of detectable peptides could be increased by improving scoring and filtering or by switching from shotgun to targeted proteomics methods or by considering PTMs.

**Figure 10:**
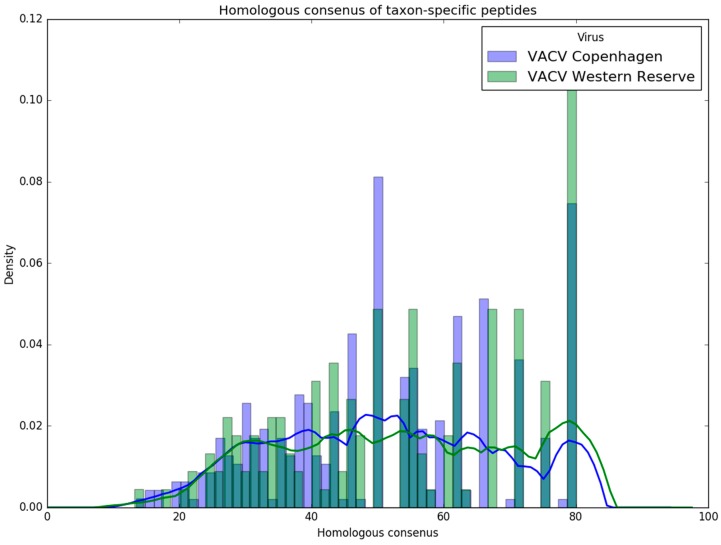
Histogram and density plot of homologous consensus. This histogram shows the distribution of the homologous consensus for the VACV Copenhagen (blue) and Western Reserve (green) strains. Additionally, the kernel density was calculated utilizing the Epanechnikov kernel and a Silverman bandwidth estimation.

Figure 10 reveals a normal distributed homologous consensus in the interval from 10% to 50%. This is caused by random matches with background peptides and these peptides should be unique for the strain. We could not observe a distinct distribution above 50%. This could be improved by moving from identity to a similarity-based matching, as this would differentiate peptides with the same amount of matching consensus residuals.

## 4. Discussion

The main goal of our developed Purple software is to provide taxon-specific peptides for a targeted proteomics assay. These targeted assays can be used in a diagnostic setting to identify a virus species/strain or even a whole virus family in a sample in sensitive and time-efficient manner. In this work, we validated the software in three different benchmarking experiments.

Purple enabled us to retrieve taxon-specific peptides to distinguish between arenavirus species proteomes that are very similar in their sequences (see Section 3.1). Accordingly, we observed a comparable decrease in the ratio of unique to *in silico*-digested peptides for New and Old World arenaviruses based on differences between their proteomes (Figure 4). This effect could also be recognized also on the clade level for the New World viruses.

The data analysis of CPXV (see Section 3.2) resulted in 56 taxon-specific peptides (Figure 7). These peptides were present in each MS/MS sample replicate and can be used to uniquely identify CPXV in a mixed biological sample, although its proteome is very similar to other Orthopoxvirus species and strains (Figure 8). By changing to a Brighton Red strain-specific target database, a reliable determination of the strain would be possible as well. This underlines that Purple relies on a correct and complete database to yield to the best possible results. Missing or incorrectly assigned protein sequences could result in incorrect selected unique peptides or discarded ones. Furthermore, although many spectra in the shotgun proteomics experiment were assigned to human peptides, this does not present a limitation for the targeted proteomics approach, because unique virus peptides selected by Purple can be detected using a targeted (e.g., PRM-based) assay in specific and sensitive manner; for example, in a recently published study [35], a PRM-based assay was used to identify dengue virus species directly from clinical serum samples. Nevertheless, to validate the resulting set of peptides, it would be recommended to test them on other CPXV samples and to check if the peptides are detectable in these samples likewise. In addition, the selected background database might be incomplete, e.g., when proteome references were missed to be included for the Purple analysis. In this case, it is useful to validate Purple-selected peptides using secondary tools such as Unipept [36] for resolving the taxonomic origin of any tryptic peptide based on the complete UniProt database. Furthermore, false negatives may result from issues during sample preparation or poor instrument performance. Therefore, these parameters need to be controlled in diagnostic PRM assays, e.g., by using internal standards and running further quality control samples.

It can be crucial in virus infection scenarios to accurately distinguish between specific strains. To cover these cases, we examined the strain-level resolution of our tool using data of VACV Copenhagen and VACV Western Reserve strains (see Section 3.3). Purple was able to find a reliable amount of strain-specific peptides (Table 7). The intersection between the Purple-selected peptides and the peptide identification from the database search showed that it is possible to detect these peptides. In general, strain-level identification was possible even for an applied FDR threshold of 1%, however, it became apparent that the shotgun proteomics approach becomes limited due to the spurious numbers of identified peptides. The number of peptides could be increased by adjusting the FDR filtering or by using a targeted proteomics approach with higher sensitivity.

In comparison to other tools, Purple offers several advantages, such as cross-platform compatibility on multiple operating systems. Purple allows a homology-based analysis of multiple proteome databases at once and produces an aggregated and summarized export on various levels. In addition, Purple is not limited to specific organisms, but can be used with general UniProt databases, also including eukaryotic and bacterial databases. High sequence similarity between strains and horizontal gene transfer may complicate taxon-specific classification for bacterial samples. However, Purple could help to overcome complications and can be helpful for creating targeted assays for bacterial detection as well. The graphical user interface and compatibility with all UniProt databases enables researchers without bioinformatics background to find taxon-specific peptides in an easy and straightforward manner.

A potential improvement to the software would be to move from a sequence identity-based metric based on the Hamming distance to similarity-based matching for the homologous matching mode. In this case, amino acid substitutions are not weighted equally, for example by using a PAM or BLOSUM matrix [37]. This similarity-based metric might allow a more accurate homologous matching in Purple. For example, an approach based on a structural alignment as introduced by Ogata et al. [38] might be useful. Further potential improvements with useful features in Purple include adding plots for better data exploration and a tabular view for inspecting the results (that are currently exportable as text files to spreadsheet software). 

In summary, the most promising application of Purple is to select taxon-specific peptides for creating tailored SRM or PRM assays with high sensitivity and specificity. This application will allow for new time- and cost-efficient diagnostic methods in healthcare and further biological applications. It could even be used to identify multiple organisms in a single sample in the context of targeted metaproteomics [39]. 

Purple is available for download on our GitLab website (https://gitlab.com/rki_bioinformatics), by using the Python package manager pip (https://pypi.org/project/purple-bio/) or via the Bioconda channel (https://anaconda.org/bioconda/purple-bio) [40]. The software is available as graphical user interface version, Python package and command line version for Windows, Linux, and MacOS. In addition, user support, tutorials, and the documentation manual can be found on the GitLab webpages.

## Figures and Tables

**Table 1:** Alphabetically ordered list of arenavirus species used for the performance benchmarking. The reader is referred to [23] for further details on these arenaviruses.

Virus Species	Abbreviation	NW/OW ^2^	NW - Clade ^3^	No. Proteins	No. Peptides ^1^
Chapare mammarenavirus	CHAV	NW	B	4	252
Guanarito mammarenavirus	GTOV	NW	B	4	244
Junin mammarenavirus	JUNV	NW	B	4	246
Lassa virus	LASV	OW	-	4	242
Lujo mammarenavirus	LUJV	OW ^4^	-	4	250
Lymphocytic choriomeningitis virus	LCMV	OW	-	4	245
Machupo virus	MACV	NW	B	4	237
Sabia mammarenavirus	SABV	NW	B	4	248
Whitewater Arroyo mammarenavirus	WWAV	NW	A/rec	4	240

^1^ Number of in silico-digested peptide sequences, ^2^ New World (NW)/ Old World (OW), ^3^ New World clade ^4^ Based on genome sequence clustering, Lujo mammaarenavirus shows its own cluster [23].

**Table 2:** This table shows the number of spectra from each sample replicate for CPXV Brighton Red, VACV Copenhagen, and VACV Western Reserve virus species/strains.

Species/Strain	No. Spectra in Replicate 1	No. Spectra in Replicate 2	No. Spectra in Replicate 3	No. Total Spectra
CPXV Brighton Red	19,396	19,352	18,920	57,668
VACV Copenhagen	19,740	19,265	19,170	58,175
VACV Western Reserve	19,421	19,453	19,076	57,950

**Table 3:** This table shows the number of taxon-specific peptides from nine arenavirus species after (i) *in silico* digest, (ii) exact matching, and (iii) homologous matching (80% background consensus threshold). Each target species was compared against the background of eight remaining arenavirus species proteomes. The second column provides the number of nonspecific peptides, i.e., the ones being shared with the background.

Species	No. Digested Peptides	No. Background Shared	No. Exact Matching	No. Homologous Matching
MACV	237	119	178	**118**
SABV	248	127	191	**121**
LUJV	250	24	241	**226**
CHAV	252	121	197	**131**
GTOV	244	75	205	**169**
JUNV	246	123	187	**123**
LASV	242	35	227	**207**
LCMV	245	31	232	**214**
WWAV	240	31	226	**209**

**Table 4:** This table shows the number of taxon-specific peptides from nine arenavirus species after (i) *in silico* digest, (ii) exact matching, and (iii) homologous matching (80% background consensus threshold). Each target species was compared against the background of eight remaining arenavirus species proteomes and additionally against all reviewed virus proteomes (from UniProt/Swiss-Prot). The second column provides the number of nonspecific peptides, i.e., the ones being shared with the background.

Species	No. Digested Peptides	No. Background Shared	No. Exact Matching	No. Homologous Matching
MACV	237	143	162	**94**
SABV	248	144	183	**104**
LUJV	250	52	229	**198**
CHAV	252	137	190	**115**
GTOV	244	118	189	**126**
JUNV	246	139	171	**107**
LASV	242	126	171	**116**
LCMV	245	110	187	**135**
WWAV	240	130	181	**110**

**Table 5:** This table shows the number of peptides from the cowpox virus (CPXV) Brighton Red strain after (i) database search with duplicates removed (CPXV); (ii) database search with duplicates removed (human); (iii) intersection of peptides obtained from Purple and peptide identifications from database search; (iv) database search, duplicates removed and filtered by 1% FDR threshold; and (iiv) intersection of peptides suggested by Purple and peptide identifications from FDR-filtered database search. The CPXV Brighton Red strain was compared against the background of all reviewed virus proteomes and the reviewed human proteome. In addition, the second column specifies the sample replicate data that was used for the database search.

Strain	Replicate	No. Database Search (CPXV)	No. Database Search (HUMAN)	No. Intersection	No. Database Search Filtered	No. Intersection Filtered
Brighton Red	1	4028	10319	94	1067	**84**
Brighton Red	2	4125	10286	83	1028	**78**
Brighton Red	3	3967	10068	92	1004	**84**

**Table 6:** This table shows the number of taxon-specific peptides from the VACV Copenhagen and VACV Western Reserve strain after (i) *in silico* digest, (ii) exact matching, and (iii) homologous matching (80% background consensus threshold). Each target strain was compared against the background of the other strain and all reviewed virus proteomes. The second column provides the number of nonspecific peptides, i.e., the ones being shared with the background.

Species	No. Digested Peptides	No. Background Shared	No. Exact Matching	No. Homologous Matching
Copenhagen	4200	3848	498	**352**
Western Reserve	4140	3971	341	**169**

**Table 7:** This table shows the number of peptides from VACV Copenhagen and VACV Western Reserve strain after (i) database search with duplicates removed; (ii) intersection of peptides obtained by Purple and database search; (iii) database search, duplicates removed and filtering by FDR; and (iv) intersection of peptides obtained by Purple and filtered database search. Each target strain was compared against the background of the other strain and all reviewed virus proteomes. The second column specifies the replicate data that was used for the database search.

Strain	Replicate	No. Database Search	No. Intersection	No. Database Search Filtered	No. Intersection Filtered
Copenhagen	1	3585	66	825	**2**
Copenhagen	2	3507	62	800	**1**
Copenhagen	3	3525	60	828	**1**
Western Reserve	1	3636	35	841	**9**
Western Reserve	2	3736	42	800	**11**
Western Reserve	3	3507	32	809	**9**

## Supplementary Materials

The following are available online at https://www.mdpi.com/1999-4915/11/6/536/s1, Table S1: Genome sequence similarities of cowpox virus; Table S2: Number of peptides from CPXV Brighton Red strain processing; Figure S1: Number of shared peptides by species for VACV Western Reserve; Figure S2: Histogram and density plot of homologous consensus—CPXV; Data S1: MSA of the pre-glycoprotein polyprotein GP complex (GPC gene); Data S2: MSA of nucleocapsid protein (N gene); Data S3: MSA of RNA-directed RNA polymerase L (L gene); Data S4: MSA of RING finger protein Z (Z gene).
