# Supplementary material for: Purple: A Computational Workflow for Strategic Selection of Peptides for Viral Diagnostics Using MS-Based Targeted Proteomics
Source: Viruses. 2019 Jun 8;11(6):536. doi: 10.3390/v11060536 (PMC6630961; doi:10.3390/v11060536)
Supplement: Supplementary file 1 [file viruses-11-00536-s001.zip › 20190315_Purple_suppl_figures_tables.docx]

**Supplementary Materials:**

**Table S1.** Genome sequence similarities of cowpox virus. The analysis was performed with blastn [https://www.ncbi.nlm.nih.gov/pmc/articles/PMC146917/]. CPXV has several highly similar viruses in the database. Ectromelia virus, as well as arumowot virus, is not endemic in humans.

| **Virus** | **Identity** | **E value** |
| --- | --- | --- |
| Vaccinia virus | 98% | 0 |
| Ectromelia virus | 97% | 0 |
| Monkeypox virus | 97% | 0 |
| Variola virus | 97% | 0 |
| Horsepox virus | 97% | 0 |

**Table S2.** This table shows the number of peptides from the CPXV Brighton Red strain after (i) database search with duplicates removed, (ii) intersection of peptides received by purple and database search, (iii) database search, duplicates removed and filtering by FDR, (iv) intersection of peptides received by purple and filtered database search. The CPXV Brighton Red strain was compared against the background of all reviewed virus proteomes. The target database consisted of all reviewed CPXV Brighton Red strain proteins. In addition, the second column specifies the replicate data that was used for the database search.

| **Strain** | **Replicate** | **No. database search** | **No. intersection** | | **No. database search filtered** | **No. intersection filtered** |
| --- | --- | --- | --- | --- | --- | --- |
| Brighton Red | 1 | 621 | | 6 | 137 | 1 |
| Brighton Red | 2 | 605 | | 6 | 132 | 1 |
| Brighton Red | 3 | 583 | | 4 | 136 | 1 |


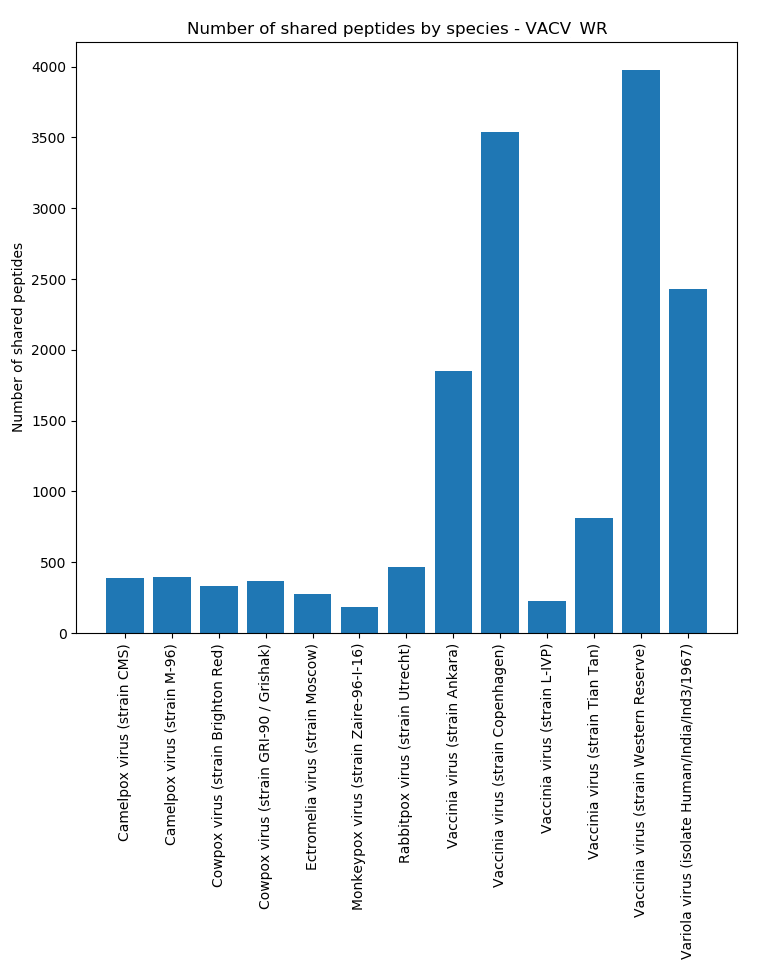


**Figure S1. Number of shared peptides by species.** This plot shows the number of shared peptides that Purple detected in the background for a species after the VACV Western Reserve analysis. All species that contribute less than 0.5% to the total amount of shared peptides were removed here.


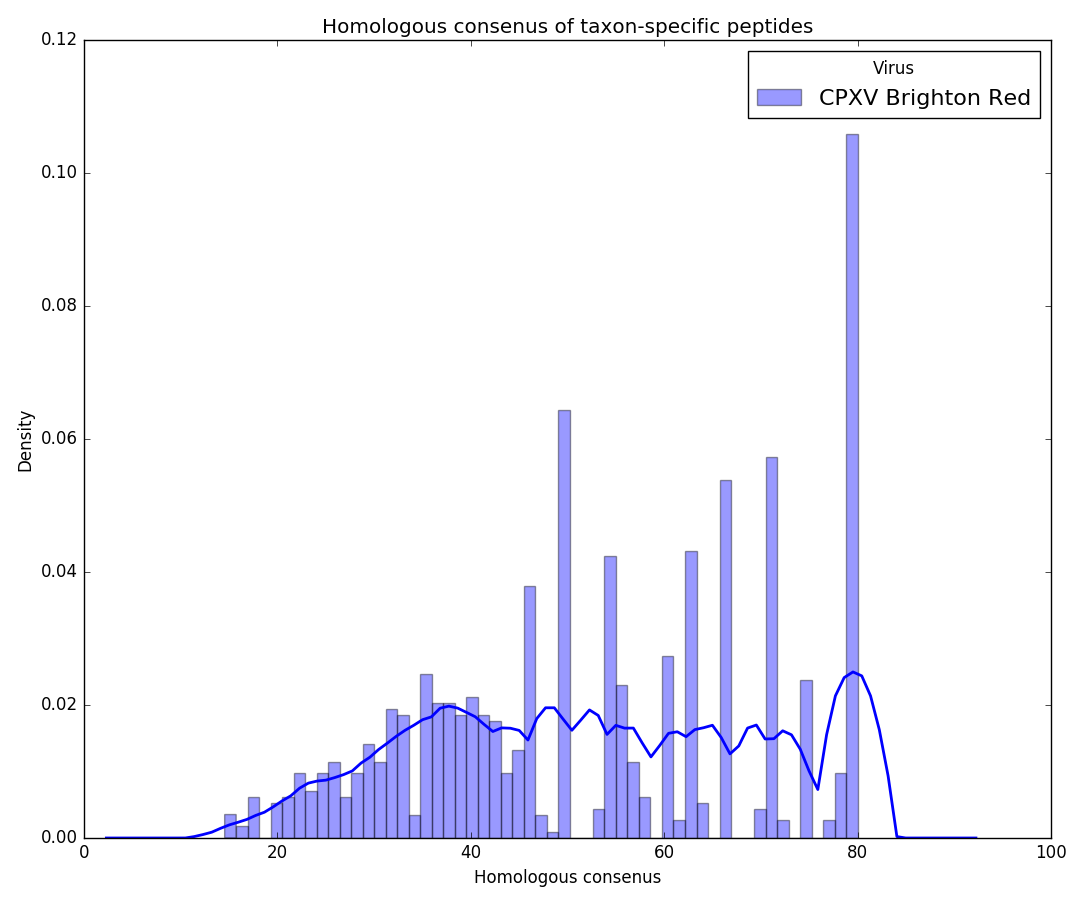


**Figure S2. Histogram and density plot of homologous consensus.** This histogram shows the distribution of the homologous consensus for the CPXV virus. Additionally, the kernel density was calculated utilizing the Epanechnikov kernel and a Silverman bandwidth estimation.
