# Supplementary material for: Purple: A Computational Workflow for Strategic Selection of Peptides for Viral Diagnostics Using MS-Based Targeted Proteomics
Source: Viruses. 2019 Jun 8;11(6):536. doi: 10.3390/v11060536 (PMC6630961; doi:10.3390/v11060536)
Supplement: Supplementary file 1 [file viruses-11-00536-s001.zip › dataS1.html]

MView


|  |
| --- |
| ``` Reference sequence (1): sp|Q911P0|GLYC_WWAVU/1480 Identities normalised by aligned length. Colored by: consensus/70% ``` |
| ```                                  cov    pid   1 [        .         .         .         .         :         .         .         . 80  1 sp|Q911P0|GLYC_WWAVU/1480   100.0% 100.0%     MGQLISFFGEIPSIIHEALNIALIAVSIISILKGVINIWGSGLLQFIVFLLLAGRSCS---YKI----GHHVELQHIILN     2 sp|P26313|GLYC_JUNIN/1485    97.8%  49.3%     MGQFISFMQEIPTFLQEALNIALVAVSLIAIIKGVVNLYKSGLFQFFVFLALAGRSCTEEAFKI----GLHTEFQTVSFS     3 sp|Q6IUF7|GLYC_MACHU/1496    97.8%  50.1%     MGQLISFFQEIPVFLQEALNIALVAVSLIAVIKGIINLYKSGLFQFIFFLLLAGRSCSDGTFKI----GLHTEFQSVTLT     4 sp|Q8AYW1|GLYC_GTOVV/1479    98.4%  48.9%     MGQLISFFQDIPIFFEEALNVALAVVTLLAIIKGIVNVWKSGILQLFVFLVLAGRSCS---FKV----GHHTNFESFTVK     5 sp|B2C4J0|GLYC_CHAVB/1484    99.5%  52.9%     MGQLVSFFQEIPNIIQEAINIALIAVSLIAILKGLVNLWKSGLFQLLVFLILAGRSCS---FKI----GRSTELQNITIN     6 sp|Q90037|GLYC_SABVB/1488    99.5%  52.7%     MGQLFSFFEEVPNIIHEAINIALIAVSLIAALKGMINLWKSGLFQLIFFLTLAGRSCS---FRI----GRSTELQNITFD     7 tr|C5ILC1|C5ILC1_9VIRU/1454  93.5%  36.9%     MGQIVAVFQAIPEILNEAINIVIIVIIMFTLIKGVFNLYKSGLFQLVIFLLLCGKRCDSSLLSG-------FNLETVHFN     8 sp|P08669|GLYC_LASSJ/1491    95.7%  42.6%     MGQIVTFFQEVPHVIEEVMNIVLIALSVLAVLKGLYNFATCGLVGLVTFLLLCGRSCT------TSLYKGVYELQTLELN     9 sp|P09991|GLYC_LYCVA/1498    96.5%  36.2%     MGQIVTMFEALPHIIDEVINIVIIVLIVITGIKAVYNFATCGIFALISFLLLAGRSCGMYGLKGPDIYKGVYQFKSVEFD       consensus/100%                                MGQhhshhttlP.hhpEshNlslhsl.hhshlKuhhNhhtsGlhthh.FLhLsG+pCs.............hphpph.hp       consensus/90%                                 MGQhhshhttlP.hhpEshNlslhsl.hhshlKuhhNhhtsGlhthh.FLhLsG+pCs.............hphpph.hp       consensus/80%                                 MGQllohFptlP.hlpEslNIsllsl.llshlKGlhNhhpsGlhthhhFLhLsGRSCs...hph....th.hphpslphs       consensus/70%                                 MGQlloFFp-lPphlpEAlNIsLlslollullKGlhNlapSGLhQhlhFLlLAGRSCo...h+h....th.hphpslphs                                       cov    pid  81          .         1         .         .         .         .         :         . 160 1 sp|Q911P0|GLYC_WWAVU/1480   100.0% 100.0%     ASYITPYVPMPCMINDTHFLLRGPFEASWAIKLEITDVTTLVVDTDN-VAPTNISKCFANNQDELGFTMEWFLH-VNAQV     2 sp|P26313|GLYC_JUNIN/1485    97.8%  49.3%     MVGLFSDLPLLCTLNKSHLYIKGGNAS---FKISFDDIAVLLPEYDVIIQPADMSWCSKSQ-IW---LSQWFMWGFIFQV     3 sp|Q6IUF7|GLYC_MACHU/1496    97.8%  50.1%     MQRLLAELPSLCMLNNSFYYMKGGVNT---FLIRVSDISVLMKEHDVSIYPEDLGNCLNKS-SW---AIHWFSWGSNIQF     4 sp|Q8AYW1|GLYC_GTOVV/1479    98.4%  48.9%     LGGVFHELPSLCRVNNSYSLIRLSHNSNQALSVEYVDVHPVLCSSSPTILD-NYTQCIKGSFDWI---LGWTIFEFTFQL     5 sp|B2C4J0|GLYC_CHAVB/1484    99.5%  52.9%     MLKVFEDHPISCTVNKTLYYIRESENATWCVEIAALDMSVLLSPHDPRVMG-NLSNCVHPDIKHLLGLLEWILFETRIQI     6 sp|Q90037|GLYC_SABVB/1488    99.5%  52.7%     MLKVFEDHPTSCMVNHSTYYVHENKNATWCLEVSVTDVTLLMAEHDRQVLN-NLSNCVHPAVEHMVGLLEWIFFETRVQI     7 tr|C5ILC1|C5ILC1_9VIRU/1454  93.5%  36.9%     MSLLSSI-PMVSE---QQHCIQHN-HSSITFSLLTNK--------S--DLKCNFTR---LQAVDFDLFRE-FHHFRVIEY     8 sp|P08669|GLYC_LASSJ/1491    95.7%  42.6%     METLNMTMPLSCTKNNSHHYIMVGNETGLELT-LTNT--------SIINHFCNLSDAHKKNLYDMSIIST-FHLFISVQY     9 sp|P09991|GLYC_LYCVA/1498    96.5%  36.2%     MSHLNLTMPNACSANNSHHYISMGT-SGLELT-FTND--------SIISHFCNLTSAFNKKTFDMSIVSS-LHLNITIQY       consensus/100%                                h.hl....P..s....p..hh..s..s...h...h.p........s......shs.....t........t.h.h...hph       consensus/90%                                 h.hl....P..s....p..hh..s..s...h...h.p........s......shs.....t........t.h.h...hph       consensus/80%                                 h.hl...hP..C.hNpohhhlp.s.ts...hp..hsc........s...h..shops.t.p......h.p.hhh.h.hQh       consensus/70%                                 M.tl..phP..ChhNpohahlphs.pus.thpl.hsD........s...h..Nhopshp.p...h..h.p.hha.hphQh                                       cov    pid 161          .         .         .         2         .         .         .         . 240 1 sp|Q911P0|GLYC_WWAVU/1480   100.0% 100.0%     NITM------EDHCSQVFMHLIVQNAYKQMIKSRTLKSFFAWSLSDATGTDMPGGYCWMLISSELKCFGNTAIAKCNMLK     2 sp|P26313|GLYC_JUNIN/1485    97.8%  49.3%     NTSK--TGINENYAKKFKLHFLTRGKN-IQLPRRSLKAFFSWSLTDSSGKDTPGGYCWMLVAAKMKCFGNTAVAKCNMLR     3 sp|Q6IUF7|GLYC_MACHU/1496    97.8%  50.1%     NISK--ADDVRVYGKKIRLHFLVRSKT-HLNFERSLKAFFSWSLTDSSGKDMPGGYCWMLIAAKMKCFGNTAVAKCNMLR     4 sp|Q8AYW1|GLYC_GTOVV/1479    98.4%  48.9%     NLTD--SPETHHYRSKIELRFLVKNAG-YLVG-RKPLAFFSWSLSDPKGNDMPGGYCWMLVAGDLKCFGNTAVAKCNMLR     5 sp|B2C4J0|GLYC_CHAVB/1484    99.5%  52.9%     NVSD--SAGSHDFKETMLLHLMMANAG-HAVKLRRLQGVFTWTITDAAGNDMPGGYCWMLVTSDLKCFGNTALAKCNMLK     6 sp|Q90037|GLYC_SABVB/1488    99.5%  52.7%     NITE--GFGSHGFEDTILMHLMLANAG-RSSGSRRPLGIFSWTITDAVGNDMPGGYCWMLVTSDLKCFGNTALAKCNMLK     7 tr|C5ILC1|C5ILC1_9VIRU/1454  93.5%  36.9%     EVTKESLPRLQEAV-STLLRLLKENSE-THYKVRKLMKLFQWSLSDETGSPLPGGHCWLIFASDIKCFDNAAIAKCNMLR     8 sp|P08669|GLYC_LASSJ/1491    95.7%  42.6%     NLSHSYAGDAANHCGTVALGLLSQRTR-DIYISRRLLGTFTWTLSDSEGKDTPGGYCWMLIEAELKCFGNTAVAKCNMLR     9 sp|P09991|GLYC_LYCVA/1498    96.5%  36.2%     NLTFSDAQSAQSQCRTFRSRILLSQEK-TKFFTRRLAGTFTWTLSDSSGVENPGGYCWMILAAELKCFGNTAVAKCNMLR       consensus/100%                                pho.......t....ph..thh.ttt.......Rp..thFtWoloD..G...PGGaCWhlhtuchKCFsNsAlAKCNML+       consensus/90%                                 pho.......t....ph..thh.ttt.......Rp..thFtWoloD..G...PGGaCWhlhtuchKCFsNsAlAKCNML+       consensus/80%                                 Nlo.......pthttphhh+hhhtptt....h.Rp.huhFsWoloDstGp-hPGGYCWMllsuchKCFGNTAlAKCNML+       consensus/70%                                 Nloc..s.tspsatpphhh+hlhpstt.phhh.RpLhuhFoWoLoDusGpDhPGGYCWMLlsu-lKCFGNTAlAKCNML+                                       cov    pid 241          :         .         .         .         .         3         .         . 320 1 sp|Q911P0|GLYC_WWAVU/1480   100.0% 100.0%     LFEFNRNQLDMIITAVNSLISDNTLKENIPYCNYTKFWYVNHTGFNVHSLPRCWLTKNYLNVSDFRNQWLLESDHLISEI     2 sp|P26313|GLYC_JUNIN/1485    97.8%  49.3%     LFDYNKNQVNLMGQTINALISDNLIRESVPYCNYTKFWYVNHTLSGQHSLPRCWLIKNYLNISDFRNDWILESDFLISEM     3 sp|Q6IUF7|GLYC_MACHU/1496    97.8%  50.1%     LFDYNKNEINLLSQTVNALISDNLIKESIPYCNYTKFWYVNHTLTGQHTLPRCWLIRNYLNTSEFRNDWILESDHLISEM     4 sp|Q8AYW1|GLYC_GTOVV/1479    98.4%  48.9%     LFDFNKNAVNMLTHSINSLISDNLLKEKVPYCNYTRFWYINHTKSGEHSLPRCWLVSNYLNESDFRNEWILESDHLIAEM     5 sp|B2C4J0|GLYC_CHAVB/1484    99.5%  52.9%     LFEFNKKKVNLLTHSINALISDNLLKEDTPYCNYTKFWYVNHTITGEHSLPRCWMVKNYLNESEFRNDWILESDHLLSEM     6 sp|Q90037|GLYC_SABVB/1488    99.5%  52.7%     LFEFNKKKVNLLTHSINALISDNLLKENTPYCNYTKFWYVNHTASGEHSLPRCWLVRNYLNESEFRNDWIIESDHLLSEM     7 tr|C5ILC1|C5ILC1_9VIRU/1454  93.5%  36.9%     LFDYNKASINLLSGRINAIISDTLLKRGIPYCNYTKFWYLNHTKLGIHSLPRCWLVSNYLNETKFTHDMEDEADKLLTEM     8 sp|P08669|GLYC_LASSJ/1491    95.7%  42.6%     LFDFNKQSIQLINKAVNALINDQLLRDGIPYCNYSKYWYLNHTTTGRTSLPKCWLVSNYLNETHFSDDIEQQADNMITEM     9 sp|P09991|GLYC_LYCVA/1498    96.5%  36.2%     LIDYNKAALHLFKTTVNSLISDQLLRDGVPYCNYSKFWYLEHAKTGETSVPKCWLVTNYLNETHFSDQIEQEADNMITEM       consensus/100%                                Lh-aN+ttlphh.ttlNulIsDphl+cthPYCNYo+aWYlpHsh.s.polP+CWhhpNYLN.ocFppph..puD.hlsEh       consensus/90%                                 Lh-aN+ttlphh.ttlNulIsDphl+cthPYCNYo+aWYlpHsh.s.polP+CWhhpNYLN.ocFppph..puD.hlsEh       consensus/80%                                 LF-aNKttlphhtpslNuLISDpLl+-shPYCNYoKFWYlNHTh.G.pSLP+CWLlpNYLN.ocFpsph..EuDphloEM       consensus/70%                                 LF-aNKpplsLlspslNuLISDsLL+-slPYCNYTKFWYlNHThoGpHSLPRCWLlpNYLNpocFps-h..EuDpLloEM                                       cov    pid 321          .         .         :         .         .         .    ] 385 1 sp|Q911P0|GLYC_WWAVU/1480   100.0% 100.0%     LSREYEARQGKTPLGLVDVCFWSTLFYVSSIFLHLLRIPTHRHIIGEACGLFKQKGRPLRWAGKV     2 sp|P26313|GLYC_JUNIN/1485    97.8%  49.3%     LSKEYSDRQGKTPLTLVDICFWSTVFFTASLFLHLVGIPTHRHIRGERCGKYPNLKKPTVWRRGH     3 sp|Q6IUF7|GLYC_MACHU/1496    97.8%  50.1%     LSKEYAERQGKTPITLVDICFWSTVFFTASLFLHLVGIPTHRHLKGERCGKYPRLRKPTIWHKRH     4 sp|Q8AYW1|GLYC_GTOVV/1479    98.4%  48.9%     LSKEYQDRQGKTPLTLVDLCFWSAIFFTTSLFLHLVGFPTHRHIQGDRCGRFQKLGKQVTWKRKH     5 sp|B2C4J0|GLYC_CHAVB/1484    99.5%  52.9%     LNKEYFDRQGKTPITLVDICFWSTLFFTTTLFLHLVGFPTHRHIQGERCGRYPELKKPTTWHRKH     6 sp|Q90037|GLYC_SABVB/1488    99.5%  52.7%     LNKEYIDRQGKTPLTLVDICFWSTLFFTTTLFLHLVGFPTHRHIRGERCGKYPELKKPITWHKNH     7 tr|C5ILC1|C5ILC1_9VIRU/1454  93.5%  36.9%     LKKEYVRRQEKTPITLMDILMFSVSFYMFSVTLCICNIPTHRHITGLACGFFKSINRSTGWAKH-     8 sp|P08669|GLYC_LASSJ/1491    95.7%  42.6%     LQKEYMERQGKTPLGLVDLFVFSTSFYLISIFLHLVKIPTHRHIVGKSCGLYKQPGVPVKWKR--     9 sp|P09991|GLYC_LYCVA/1498    96.5%  36.2%     LRKDYIKRQGSTPLALMDLLMFSTSAYLVSIFLHLVKIPTHRHIKGGSCGAFKVPGVKTVWKRR-       consensus/100%                                Lp+-Y.tRQtpTPlsLhDlhhaSs.hah.olhLplhthPTHRHl.G.tCGha...th.hhWtt..       consensus/90%                                 Lp+-Y.tRQtpTPlsLhDlhhaSs.hah.olhLplhthPTHRHl.G.tCGha...th.hhWtt..       consensus/80%                                 LpKEY.cRQGKTPlsLhDlhhaSs.FahholFLHLlthPTHRHI.GttCGha.p.th.hhWt+t.       consensus/70%                                 LpKEY.cRQGKTPlsLVDlhhaST.FahsSlFLHLVthPTHRHIpGcpCGha.pht+sshW++p. ``` |

MView 1.63, Copyright © 1997-2018 Nigel P. Brown
