# Supplementary material for: Purple: A Computational Workflow for Strategic Selection of Peptides for Viral Diagnostics Using MS-Based Targeted Proteomics
Source: Viruses. 2019 Jun 8;11(6):536. doi: 10.3390/v11060536 (PMC6630961; doi:10.3390/v11060536)
Supplement: Supplementary file 1 [file viruses-11-00536-s001.zip › dataS2.html]

MView


|  |
| --- |
| ``` Reference sequence (1): tr|C5ILC2|C5ILC2_9VIRU/1558 Identities normalised by aligned length. Colored by: consensus/70% ``` |
| ```                                  cov    pid   1 [        .         .         .         .         :         .         .         . 80  1 tr|C5ILC2|C5ILC2_9VIRU/1558 100.0% 100.0%     MSQSKEVKSFLWLQTLRRELSPFCTDVRAKVINGLDFSMVSDVQRLMRKDKRNDEDLMKLRELNQTVDGLVDLKSSNKKN     2 sp|P13699|NCAP_LASSJ/1569   100.0%  59.3%     MSASKEIKSFLWTQSLRRELSGYCSNIKLQVLHGLDFSEVSNVQRLMRKERRDDNDLKRLRDLNQAVNNLVELKSTQQKS     3 sp|P09992|NCAP_LYCVA/1558    99.3%  60.9%     MSLSKEVKSFQWTQALRRELQSFTSDVKAAVLNGLDFSEVSNVQRIMRKEKRDDKDLQRLRSLNQTVHSLVDLKSTSKKN     4 sp|Q91PB2|NCAP_WWAVU/1562    99.8%  47.9%     -MSDQSVPSFRWTQSLRRGLSAWTTSVKADVLSGLDFAKVASVQRMMRRVKRDDSDLVGLRDLNKEVDSLMIMKSNQKNM     5 sp|B2C4J1|NCAP_CHAVB/1562    99.8%  48.5%     MSNSKEIPSFRWTQSLRRELSSFTIPVKSDVADGLDFSQVALVQRVLRKTKRTDGDLDKLRDLNREVDNLMAMKSAQKNT     6 sp|Q90038|NCAP_SABVB/1562    99.8%  49.8%     MSNSKEIPSFRWTQSLRRGLSEFTTPVKTDVLDGLDFNQVSLVQRILRKSKRNDGDLDKLRDLNKEVDNLMSMKSSQRDT     7 sp|Q8AZ66|NCAP_GTOVV/1560    99.6%  48.0%     MAHSKEIPSFRWTQSLRRELGMFTEPTKSSVADSLDFTQVSQVQRLLRKSKRGDTDLDKLRDLNKEVDRLMSMKSVQNNT     8 sp|P14239|NCAP_JUNIN/1564    99.6%  45.9%     MAHSKEVPSFRWTQSLRRGLSQFTQTVKSDVADSIDFNQVAQVQRALRKTKRGEEDLNKLRDLNKEVDRLMSMRSVQRNT     9 sp|P26578|NCAP_MACHU/1564    99.6%  47.6%     MAHSKEIPSFRWTQSLRRGLSQVHPTVKTDVADSIDFNQVSQVQRALRKNKRGEEDLNKLRDLNKEVDRLMSMKSIQKNT       consensus/100%                                ...sppl.SF.WhQsLRRtLt.hp.sh+.tVhpulDFs.Vu.VQRhhR+.+Rs-tDL.tLRpLNptVptLh.h+S.ppp.       consensus/90%                                 ...sppl.SF.WhQsLRRtLt.hp.sh+.tVhpulDFs.Vu.VQRhhR+.+Rs-tDL.tLRpLNptVptLh.h+S.ppp.       consensus/80%                                 MutSKEl.SFhWTQoLRRtLu.as.slKspVhsulDFspVu.VQRhhRKpKRs-tDL.+LR-LNppVcpLhthKSsppps       consensus/70%                                 MupSKEl.SFpWTQSLRRtLStaspsVKssVhsuLDFspVupVQRhhRKsKRsDpDLp+LRDLNppVDpLhshKSsQ+ps                                       cov    pid  81          .         1         .         .         .         .         :         . 160 1 tr|C5ILC2|C5ILC2_9VIRU/1558 100.0% 100.0%     RVGVGKLTSDELMILATDLEKLKKKVTRTEA--RGPGVYRGNLSQDQLGRRSELLNMIGMGTPRP----TRNTVVRVWDV     2 sp|P13699|NCAP_LASSJ/1569   100.0%  59.3%     ILRVGTLTSDDLLILAADLEKLKSKVIRTERP-LSAGVYMGNLSSQQLDQRRALLNMIGMSGGNQGARAGRDGVVRVWDV     3 sp|P09992|NCAP_LYCVA/1558    99.3%  60.9%     VLKVGRLSAEELMSLAADLEKLKAKIMRSERP-QASGVYMGNLTTQQLDQRSQILQIVGMRKPQQ----GASGVVRVWDV     4 sp|Q91PB2|NCAP_WWAVU/1562    99.8%  47.9%     FLKVGSLSKDELMELSSDLEKLKQKVQRTERVGNGTGQYQGNLSNTQLTRRSEILQLVGIQRAGL---APTGGVVKIWDI     5 sp|B2C4J1|NCAP_CHAVB/1562    99.8%  48.5%     ILKLGDLNKSELMDLASDLEKLKKKVGQTERS-PVGGVYLGNLSQSQLSKRTDLLRRLGFQQPQV---R-STGVVRIWDV     6 sp|Q90038|NCAP_SABVB/1562    99.8%  49.8%     ILKLGDLNKSELMDLASDLEKLKRKVGQTERS-ASGGVYLGNLSQSQLTKRSDLLRKLGFQQQQV---R-SPGVVRIWDV     7 sp|Q8AZ66|NCAP_GTOVV/1560    99.6%  48.0%     VLKVGDLGKDELMDLASDLEKLKKKIGDRESN--SPRMYMGNLTQSQLEKRAGILRTLGFQQQRG---A-AGGVVRLWDV     8 sp|P14239|NCAP_JUNIN/1564    99.6%  45.9%     VFKAGDLGRVERMELASGLGNLKTKFRRAETG--SQGVYMGNLSQSQLAKRSEILRTLGFQQQGT---G-GNGVVRVWDV     9 sp|P26578|NCAP_MACHU/1564    99.6%  47.6%     IFKIGDLGRDELMELASDLEKLKNKIKRTESG--PQGLYMGNLSQLQLTKRSEILKTLGFQQQRG---A-GNGVVRIWDV       consensus/100%                                hhthGpLst.-hh.LussLtpLKtKh.ptEt...s.t.Y.GNLop.QLtpRttlLphlGhtt.t......tssVV+lWDl       consensus/90%                                 hhthGpLst.-hh.LussLtpLKtKh.ptEt...s.t.Y.GNLop.QLtpRttlLphlGhtt.t......tssVV+lWDl       consensus/80%                                 hh+lGpLspsELM.LAuDLEKLKpKlhpsEps..s.GhYhGNLoppQLspRstlLphlGhpp.t......tsGVVRlWDV       consensus/70%                                 llKlGcLspsELMpLAoDLEKLKpKltcoEps..usGlYhGNLSppQLs+RoplLphlGhpp.p....t.ssGVVRlWDV                                       cov    pid 161          .         .         .         2         .         .         .         . 240 1 tr|C5ILC2|C5ILC2_9VIRU/1558 100.0% 100.0%     KDSSLLNNQFGTMPSLTLACLTRQTRVDLNDSVQACVDLGLIYTAKFPNMDDLDKLKNKHPVLDYVSNCDSAINISGYNL     2 sp|P13699|NCAP_LASSJ/1569   100.0%  59.3%     KNAELLNNQFGTMPSLTLACLTKQGQVDLNDAVQALTDLGLIYTAKYPNTSDLDRLTQSHPILNMIDTKKSSLNISGYNF     3 sp|P09992|NCAP_LYCVA/1558    99.3%  60.9%     KDSSLLNNQFGTMPSLTMACMAKQSQTPLNDVVQALTDLGLLYTVKYPNLNDLERLKDKHPVLGVITEQQSSINISGYNF     4 sp|Q91PB2|NCAP_WWAVU/1562    99.8%  47.9%     KDPSLLVNQFGSVPAVTISCMTEQGGESLNDVVQGLTDLGLLYTAKYPNLNDLKALTTKHPSLNIITQEESQINISGYNL     5 sp|B2C4J1|NCAP_CHAVB/1562    99.8%  48.5%     ADPTRLNNQFGSVPALTIACMTVQGGDTMGNVVQALTSLGLLYTVKFPNLADLEKLAAEHDCLQIITKDESAINISGYNF     6 sp|Q90038|NCAP_SABVB/1562    99.8%  49.8%     ADPNRLNNQFGSVPALTIACMTKQSDNTMGDVVQALTSLGLLYTVKFPNLIDLEKLTAEHDCLQIVTKDESGLNISGYNY     7 sp|Q8AZ66|NCAP_GTOVV/1560    99.6%  48.0%     SDPSKLNNQFGSMPALTIACMTVQGGETMNNVVQALTSLGLLYTVKYPNLDDLEKLTLEHDCLQIITKDESALNISGYNF     8 sp|P14239|NCAP_JUNIN/1564    99.6%  45.9%     KDPSKLNNQFGSVPALTIACMTVQGGETMNSVIQALTSLGLLYTVKYPNLSDLDRLTQEHDCLQIVTKDESSINISGYNF     9 sp|P26578|NCAP_MACHU/1564    99.6%  47.6%     SDPSKLNNQFGSMPALTIACMTVQGGETMNSVVQALTSLGLLYTVKYPNLNDLDKLTLEHECLQIVTKDESSINISGYNF       consensus/100%                                tssphLsNQFGohPulThuChs.Qst.shssslQuhssLGLlYTsKaPNh.DLctLt.pH..LthlspppStlNISGYNh       consensus/90%                                 tssphLsNQFGohPulThuChs.Qst.shssslQuhssLGLlYTsKaPNh.DLctLt.pH..LthlspppStlNISGYNh       consensus/80%                                 tDsshLNNQFGohPuLTlAChThQut.shsssVQALTsLGLlYTsKaPNhsDL-+Lp.cHssLphloppcSulNISGYNh       consensus/70%                                 pDsohLNNQFGohPuLTlACMThQutpshNsVVQALTsLGLLYTsKaPNLsDL-+LstcHssLpllTpc-SulNISGYNa                                       cov    pid 241          :         .         .         .         .         3         .         . 320 1 tr|C5ILC2|C5ILC2_9VIRU/1558 100.0% 100.0%     SLASLVKAGSRNIDDVIKATLTARNKVQMFVSEVPGERNPYENLLYKICLSGEGWPYISSRTSIKGRSWDNTVIDMTP--     2 sp|P13699|NCAP_LASSJ/1569   100.0%  59.3%     SLGAAVKAGPQTMDGILKSILKVKKALGMFISDTPGERNPYENILYKICLSGDGWPYIASRTSITGRAWENTVVDLESDG     3 sp|P09992|NCAP_LYCVA/1558    99.3%  60.9%     SLGAAVKAGPSNSEDLLKAVLGAKRKLNMFVSDQVGDRNPYENILYKVCLSGEGWPYIACRTSIVGRAWENTTIDLTSE-     4 sp|Q91PB2|NCAP_WWAVU/1562    99.8%  47.9%     SLSAAVKAGESTFTTVIKTLLEVKNKEKMFVSPTPGQRNPYENVLYKLCLSGDGWPYIASRSQIKGRAWDNTVVEFDT--     5 sp|B2C4J1|NCAP_CHAVB/1562    99.8%  48.5%     SLSAAVKAGPDNFSTIIKTVLGVKKRENMFIDERPGNRNPYENLLYKLCLSGEGWPYIGSRSQVKGRSWENTTVDLSL--     6 sp|Q90038|NCAP_SABVB/1562    99.8%  49.8%     SLSAAVKAGPDNFSQIIKTTLSIKKKEGMFVDEKPGNRNPYENLLYKICLSGEGWPYIGSRSQIKGRSWENTTVDLST--     7 sp|Q8AZ66|NCAP_GTOVV/1560    99.6%  48.0%     SLSAAVKAGPNNFSSIVKAALNVKRREGMFIDERPGNRNPYENLLYKLCLSGEGWPYIGSRSQILGRSWDNTSVDLNA--     8 sp|P14239|NCAP_JUNIN/1564    99.6%  45.9%     SLSAAVKADPDNFSSLIKSTIQVKRREGMFIDEKPGNRNPYENLLYKLCLSGDGWPYIGSRSQIIGRSWDNTSIDLTR--     9 sp|P26578|NCAP_MACHU/1564    99.6%  47.6%     SLSAAVKAGPDNFSSLIKSTLQVKRKEGMFIDEKPGNRNPYENLLYKLCLSGDGWPYIGSRSQILGRSWDNTSVDLTK--       consensus/100%                                SLuuhVKAs.ps.ptllKshlth+pt.tMFls..sGpRNPYENlLYKlCLSG-GWPYIusRoplhGRuW-NTsl-hp...       consensus/90%                                 SLuuhVKAs.ps.ptllKshlth+pt.tMFls..sGpRNPYENlLYKlCLSG-GWPYIusRoplhGRuW-NTsl-hp...       consensus/80%                                 SLuAAVKAGspshssllKshLtsKp+.tMFls-pPGpRNPYENlLYKlCLSG-GWPYIuSRopIhGRuW-NTslDhs...       consensus/70%                                 SLuAAVKAGPsNhssllKssLplK++.sMFls-pPGpRNPYENlLYKlCLSG-GWPYIuSRopIhGRuW-NTslDLst..                                       cov    pid 321          .         .         :         .         .         .         .         4 400 1 tr|C5ILC2|C5ILC2_9VIRU/1558 100.0% 100.0%     --KDPTPPQGVSFSQSQLLDDIMKNLNPKGRTWMDIEGRPDDPVEIAIFQFYRTDQKQGMDFTQLCSTQPGLTTAVLERL     2 sp|P13699|NCAP_LASSJ/1569   100.0%  59.3%     KPQKADSNNGLTYSQLMTLKDAMLQLDPNAKTWMDIEGRPEDPVEIALYQFFRTDLKQGIDVTDLFATQPGLTSAVIDAL     3 sp|P09992|NCAP_LYCVA/1558    99.3%  60.9%     KPAVNSPRPGLSYSQTMLLKDLMGGIDPNAPTWIDIEGRFNDPVEIAIFQFYRVDQKQGMDLADLFNAQPGLTSSVIGAL     4 sp|Q91PB2|NCAP_WWAVU/1562    99.8%  47.9%     ATVKEPIPITLKPEIENQVKRSVESLLINDTTWIDIEGPPNDPVEFAIYQCYRNDIKSGILLKDVENARPGLISSIIRSL     5 sp|B2C4J1|NCAP_CHAVB/1562    99.8%  48.5%     KPTQGPKAPHLTEIQESVVREAMSKINPSHTTWIDIEGTSNDPVELALYQCYRHDEKGGMLLKDLESAQPGLLSYIIGLL     6 sp|Q90038|NCAP_SABVB/1562    99.8%  49.8%     KPQQGPRTPHLTELQESVVREAMGKIDPTLTTWIDIEGTSNDPVELALYQCYRHDEKGGMLLKDLESAQPGLLSYVIGLL     7 sp|Q8AZ66|NCAP_GTOVV/1560    99.6%  48.0%     RPVTGPRAPNLSEMQEAIVKEAMRKLDSSDTIWMDIEGPPTDPVELAVFQCFRHDEKGGILLKDLEDAQPGLLSYVIGLL     8 sp|P14239|NCAP_JUNIN/1564    99.6%  45.9%     KPVAGPRQPNLTEIQEAVIREAVGKLDPTNTLWLDIEGPATDPVEMALFQCFRHDEKGGILMKDIEDAMPGVLSYVIGLL     9 sp|P26578|NCAP_MACHU/1564    99.6%  47.6%     KPQVGPRQPNLTEMQEAVIKEAVKKLDPTNTLWLDIEGPPTDPVELALYQCFRHDEKGGILMQDIEDAMPGVLSYVIGLL       consensus/100%                                .....s...tlp......lcc.h.tl..p..hWhDIEG..pDPVEhAlaQhaR.D.KtGh.htpl.ss.PGlho.llt.L       consensus/90%                                 .....s...tlp......lcc.h.tl..p..hWhDIEG..pDPVEhAlaQhaR.D.KtGh.htpl.ss.PGlho.llt.L       consensus/80%                                 ts..ss...tLo..Qp.hl+-hhtplssstphWhDIEG.ssDPVEhAlaQhaRpD.KtGh.hpDl.ss.PGlhS.lIthL       consensus/70%                                 +P.tss.tssLo..Qptll+-uhtplsPssshWhDIEG.ssDPVElAlaQhaRpDpKtGh.hpDlpsApPGLhShVIshL                                       cov    pid 401          .         .         .         .         :         .         .       ] 478 1 tr|C5ILC2|C5ILC2_9VIRU/1558 100.0% 100.0%     PLGMVIEKEASRKFEDQVWDSYKTFCNQHTGIVVTKSKK-GK-----KEITPHCALMDCRNLLPADMIFRTAAKLSL-     2 sp|P13699|NCAP_LASSJ/1569   100.0%  59.3%     PRNMVIRKTDSRKYENAVWDQYKDLCHMHTGVVVEKKKRGGK-----EEITPHCALMDCRAVLPRDMVFRTSTPRVVL     3 sp|P09992|NCAP_LYCVA/1558    99.3%  60.9%     PQGMVIRKEASREYEDKVWDKYGWLCKMHTGIVRDKK----K-----KEITPHCALMDCHNILPHDLIFRGPNVVTL-     4 sp|Q91PB2|NCAP_WWAVU/1562    99.8%  47.9%     PKSMVIRKEESRIFEDLVWKRFEHLCDKHKGIVIKSKKKGSTP----ATTNAHCALLDGKRMLPIDLLFREPETTVVL     5 sp|B2C4J1|NCAP_CHAVB/1562    99.8%  48.5%     PQDMVIKRDQARNYEEQVWSDFGHLCKKHNGVVVPKKKKDKDPS---QSTEPHCALLDCQSLLPEVLLFTMKPAFAI-     6 sp|Q90038|NCAP_SABVB/1562    99.8%  49.8%     PQNMVIRREQARKFEEPIWSDFGHLCKKHNGVIVPKKKKDKDIP---QSSEPHCALLDCEGLLPDALLFTLEAAFTI-     7 sp|Q8AZ66|NCAP_GTOVV/1560    99.6%  48.0%     PQGSVIKKEQSRIFEQEVWEKFGHLCRAHNGVIVPKKKNKEA----NSTKEPHCALLDCIQLLPNTLVFQAKSAFVM-     8 sp|P14239|NCAP_JUNIN/1564    99.6%  45.9%     PPDMVIRKEQARQFDQQVWEKFGHLCKHHNGVVVSKKKRDKDAPFKLASSEPHCALLDCTPLLPPSLLFLPKAAYAL-     9 sp|P26578|NCAP_MACHU/1564    99.6%  47.6%     PQDMVIRKDQARLYDQQIWEKFGHLCKHHNGVVVNKKKREKDSPFKLSSGEPHCALLDCVALLPLSLLFLPKAAFAL-       consensus/100%                                P.s.VIc+ptuR.a-p.lWppat.hCp.HpGllh.pp....t.....tp.psHCALhDs..hLP.shlF.....hsh.       consensus/90%                                 P.s.VIc+ptuR.a-p.lWppat.hCp.HpGllh.pp....t.....tp.psHCALhDs..hLP.shlF.....hsh.       consensus/80%                                 P.sMVI++-tuR.a-p.lWppatpLCchHsGlll.KKKp.tp.....tp.pPHCALhDC.tlLP.shlFh..sshsl.       consensus/70%                                 PpsMVI+K-puR.aEptVWccataLC+tHsGlVVsKKK+ttc.....pphpPHCALhDCpslLP.sLlFp.tsshsl. ``` |

MView 1.63, Copyright © 1997-2018 Nigel P. Brown
