# Supplementary material for: Purple: A Computational Workflow for Strategic Selection of Peptides for Viral Diagnostics Using MS-Based Targeted Proteomics
Source: Viruses. 2019 Jun 8;11(6):536. doi: 10.3390/v11060536 (PMC6630961; doi:10.3390/v11060536)
Supplement: Supplementary file 1 [file viruses-11-00536-s001.zip › dataS3.html]

MView


|  |
| --- |
| ``` Reference sequence (1): sp|B2ZDY2|L_WWAVU/12219 Identities normalised by aligned length. Colored by: consensus/70% ``` |
| ```                                   cov    pid    1 [        .         .         .         .         :         .         .         . 80   1 sp|B2ZDY2|L_WWAVU/12219      100.0% 100.0%      -MVDCNDRINELKDLIRKWVPDEEAYTEQKTTFLSQVNPSSIITEGLKLLSMLIEIDSCLKHGCVFNRN-KTVNQILKDH      2 sp|Q6UY70|L_GTOVV/12198       97.6%  43.1%      ----MDEKVFVLKDFIRRQVPDIPELSYQKEALLSQVEVPMVLTEGFKLLSCLVEIESCRKNSCECNFEQKFVDTILSEN      3 sp|Q6XQI4|L_JUNIN/12210       98.1%  43.2%      ----MEESVNEIKNLIRKHFPERQELAYQRDIFLSQHHPSSLLLEGFKLLSSLVELESCEAHACQINSDQKFVDVILSDH      4 sp|Q6IUF8|L_MACHU/12209       98.0%  43.5%      ----MDEYVQELKGLIRKHIPDRCEFAHQKVTFLSQVHPSPLLTEGFKLLSSLVELESCEAHACQANTDQRFVDVILSDN      5 sp|B2C4J3|L_CHAVB/12208       98.0%  42.4%      ----MDTFLLELKDLVRKYVPELVELSFQKDALLSQVHPRLVLVEGFKLLSLLVELESCKVNACRHNFEQKFVDVILSDH      6 sp|Q6UY61|L_SABVB/12212       98.1%  42.0%      MQDPLLGTLSELKDLVRKTIPDVIELAYQKDALLSQVHPRSVLIEGFKLLSLLVELESCKVNACHHNYEQKFIDVILSDG      7 tr|C5ILC4|C5ILC4_9VIRU/12234  95.9%  33.1%      ----MDSLKNELLQLISRNFPNDERLAPQKLKVLCQTEPKFIMIEGLKLLSTCIEIDICDENGCIHNTEEKSVELILREN      8 sp|O09705|L_LASSJ/12218       96.1%  33.7%      ----MEEDIACVKDLVSKYLVDNERLSRQKLAFLVQTEPRMLLMEGLKLLSLCIEVDSCNANGCEHNSEDKSVERILHDH      9 sp|P14240|L_LYCVA/12210       95.7%  33.3%      ----MDEIISELRELCLNYIEQDERLSRQKLNFLGQREPRMVLIEGLKLLSRCIEIDSADKSGCTHNHDDKSVETILVES        consensus/100%                                  ....h.t.h..lhthh.p.h.p..ths.Q+..hLsQhps..lhhEGhKLLS.hlEl-.s..puC.hN.p.+.lp.IL.-t        consensus/90%                                   ....h.t.h..lhthh.p.h.p..ths.Q+..hLsQhps..lhhEGhKLLS.hlEl-.s..puC.hN.p.+.lp.IL.-t        consensus/80%                                   ....hpp.l.pl+pLlp+.hsp..chu.QK.thLsQhcPp.llhEGhKLLS.hlEl-SCphpuC.hN.-pK.V-hILp-p        consensus/70%                                   ....M-phltELKsLlp+hlP-..cLuhQK.shLuQscPp.lLhEGhKLLShhlEl-SCctpuCptNh-pK.V-hILp-p                                         cov    pid   81          .         1         .         .         .         .         :         . 160  1 sp|B2ZDY2|L_WWAVU/12219      100.0% 100.0%      RIIGPTLPDVVPDGYRVSGSTIILLETFVRVNQESFEQKYKHDFEKLIQLSKDLSKCGLILVPVIDGRSNYYVDRFPDWV      2 sp|Q6UY70|L_GTOVV/12198       97.6%  43.1%      GVVAPTLPKVIPDGYRFFNKTLILLETFVRVNPEEFEKKWKTDMAKLLSLKEDIHRTGITLVPVVDGRGNYNTDLLPDWA      3 sp|Q6XQI4|L_JUNIN/12210       98.1%  43.2%      GILCPTLPKVIPDGFKLTGKTLILLETFVRVNPDEFEKKWKSDMSKLLNLKSDLLRAGITLVPVVDGRSSYSNRFLADWV      4 sp|Q6IUF8|L_MACHU/12209       98.0%  43.5%      GILCPTLPKVIPDGFKLTGKTLILLETFVRVNPDEFEKKWKADMSKLLNLKHDLQKSGVTLVPIVDGRSNYNNRFVADWV      5 sp|B2C4J3|L_CHAVB/12208       98.0%  42.4%      GVICPTLPKVTPDGFNLMGKTLILLETFVRVNPNDFERKWKADMSKLMSLKDDLARVGITMVPVVDGRGSYNTSYLPEWA      6 sp|Q6UY61|L_SABVB/12212       98.1%  42.0%      GILCPTLPKVVPDGYNLMGKTLILLETFVRVNPDDFEKKWKADMSKLISLKTDLGKIGVTLVPVVDGRSNYNTSFVSDWT      7 tr|C5ILC4|C5ILC4_9VIRU/12234  95.9%  33.1%      GFVVPSLPCVTPDGFKVSGNMLILLECFVRLNPLEYEQKYNNDMKKLSSLRGDLEHSGITLVPLVVGRSGYENNSIPDWV      8 sp|O09705|L_LASSJ/12218       96.1%  33.7%      GILTPSLCLWYPDGYKLTGNVLILLECFVRSSPANFEQKYIEDFKKLEQLKEDLKSVDINLIPLIDGRTSFYNEQIPDWV      9 sp|P14240|L_LYCVA/12210       95.7%  33.3%      GIVCPGLPLIIPDGYKLIDNSLILLECFVRSSPASFEKKFIEDTNKLACIREDLAVAGVTLVPIVDGRCDYDNSFMPEWA        consensus/100%                                  thlsPsLs.hhPDGaph.sp.lILLEsFVR.s..paEpKa.tDhtKL.plptDl...sl.hlPllsGRssa.sp.hs-Ws        consensus/90%                                   thlsPsLs.hhPDGaph.sp.lILLEsFVR.s..paEpKa.tDhtKL.plptDl...sl.hlPllsGRssa.sp.hs-Ws        consensus/80%                                   GllsPoLPhlhPDGapl.spsLILLEsFVRssPtpFEpKahtDhpKL.pL+pDLtpsGlsLVPllDGRssY.sp.hs-Ws        consensus/70%                                   GllsPoLPpVhPDGa+lhGpoLILLEsFVRlNPtpFEpKaptDhpKLhsL+pDLt+sGlTLVPlVDGRusYpsphlsDWs                                         cov    pid  161          .         .         .         2         .         .         .         . 240  1 sp|B2ZDY2|L_WWAVU/12219      100.0% 100.0%      VERIRWLLLKLMDNVKDTGE-RIEELEYNRLISSLSSMENQNLGLESLKALREEGLDYKTKLMEAIRDGIRPNLTASECR      2 sp|Q6UY70|L_GTOVV/12198       97.6%  43.1%      TERFRWLLIDLLRESRGAPTMEIEDQEYHRLIHSLSKTSNQSLGFENIECLKRVHLNYEERLNEQLLKDIVGEVRESKIR      3 sp|Q6XQI4|L_JUNIN/12210       98.1%  43.2%      VERVRWLLIDILKKSKFMQEINIEEQEYQRLIHSLSNTKNQSLGLENIECLKKNSLGYDERLNESLFVGVRGDIRESVIR      4 sp|Q6IUF8|L_MACHU/12209       98.0%  43.5%      IERMRWLLIEILKASKSMLEIDIEDQEYQRLIHSLSNVKNQSLGLENLEHLKRNSLDYDERLNESLFIGLKGDIRESTVR      5 sp|B2C4J3|L_CHAVB/12208       98.0%  42.4%      TERLRWLLIEILKGVKATSEIEIEDQEYQRLIHSLAKANNQSMGFENLEFLKRRLLSYDQLLDTSLLVGIRNDVRESKII      6 sp|Q6UY61|L_SABVB/12212       98.1%  42.0%      TERLRWLLIEVLKGMKTTSELEIEEQEYHRLIHSLAKTNNQSLGFENLECLKRNMLSYDQLLDSSLLVGVKNDVKESKVM      7 tr|C5ILC4|C5ILC4_9VIRU/12234  95.9%  33.1%      VFKFKNLIFRLLEYVQEN-EAMLEESEYIRLSESLKGNGSKLFDLDAFNLLKNNSGEHFENIMRLCVEGVRPDIPLSEVN      8 sp|O09705|L_LASSJ/12218       96.1%  33.7%      NDKLRDTLFSLLKYAQES-NSLFEESEYSRLCESLFMTSGRLSGVESLNVLMDNRSNHYEEVIASCHQGINNKLTAHEVK      9 sp|P14240|L_LYCVA/12210       95.7%  33.3%      NFKFRDLLFKLLEYSNQN-EKVFEESEYFRLCESLKTTIDKRSGMDSMKILKDARSTHNDEIMRMCHEGINPNMSCDDVV        consensus/100%                                  ..+h+.hlhplhc..p...p..hE-.EY.RL.pSL....sp..sh-shphLhp...ta.p.l.t.hh.sl.sph..p.h.        consensus/90%                                   ..+h+.hlhplhc..p...p..hE-.EY.RL.pSL....sp..sh-shphLhp...ta.p.l.t.hh.sl.sph..p.h.        consensus/80%                                   s.+hR.LLhclLc.spt..p..hE-pEY.RLhcSLttstsp..Gh-shphL+ct..sa.p.l.p.hh.Glpsplptsplh        consensus/70%                                   s-+hR.LLhclLchspts.Eh.lE-pEYpRLhcSLtpspspphGhEslchLKcsthsapppl.pthh.GlpsclptSclh                                         cov    pid  241          :         .         .         .         .         3         .         . 320  1 sp|B2ZDY2|L_WWAVU/12219      100.0% 100.0%      IGIAKVY---DQFCLLRDSGQYQNVYCRTSRSEMIEWLKDHKLTSLINGSEGTFFNNERCGFCQNHMLRVIAELVHSKRV      2 sp|Q6UY70|L_GTOVV/12198       97.6%  43.1%      EELIKLKTWYREEIYRKGLGNF----VQTDRKSLL--------QTLVLSSAHSDSLAPECPMCCSKILDLCYQLSMRIAN      3 sp|Q6XQI4|L_JUNIN/12210       98.1%  43.2%      EELIKLRFWFKKEIFDKQLGKF----KFSQKSNLI--------NDLVSLGSHKDSDVPSCPFCANKLMDVVYSIASHPID      4 sp|Q6IUF8|L_MACHU/12209       98.0%  43.5%      EELIKLKMWFKDEVFSKGLGKF----KLTDRRELL--------ESLSSLGAHLDSDVSSCPFCNNKLMEIVYNVTFSSVE      5 sp|B2C4J3|L_CHAVB/12208       98.0%  42.4%      EELIKIKLWYKTEIFNKGLGKF----KRTNKSNLL--------SDLLKIGLHQDSDTINCMFCSCKILELCYTLSNKLSI      6 sp|Q6UY61|L_SABVB/12212       98.1%  42.0%      EELIRLKIWYKSEVYEKGLGKF----VKTDKKVLL--------SQLITLGSHEENDSLDCAFCSSRILELCFKLSVKMHE      7 tr|C5ILC4|C5ILC4_9VIRU/12234  95.9%  33.1%      SKIVENFLEFKAKL---KKGVIPPLFMMTDKERLLKEFCDLYLDQGVSSAPESKWELLRDFKEATSFTNFIYFDSKND--      8 sp|O09705|L_LASSJ/12218       96.1%  33.7%      LQIEEEYQVFRNRL---RKGEIEGQFLKVDKSQLLNELNNLYADKVVAE--DNIEHLIYQFKRASPILRFLYANVDEG--      9 sp|P14240|L_LYCVA/12210       95.7%  33.3%      FGINSLFSRFRRDL---ESGKLKRNFQKVNPEGLIKEFSELYENLADS---DDILTLSREAVESCPLMRFITAETHG---        consensus/100%                                  .tl.p.h...ct.h...t.G.h......sp.p.hl........p.h.....t.......p.hpts.hhphhh..s.t...        consensus/90%                                   .tl.p.h...ct.h...t.G.h......sp.p.hl........p.h.....t.......p.hpts.hhphhh..s.t...        consensus/80%                                   .tl.c.h..a+pcl...t.Gph....hhss+ptLl........pph.t...cp..p..pp.hpss.hhchhht.s.p...        consensus/70%                                   .plhclh.ha+pcl...thGph....hhos+ppLl........splls.u.cp..ph.ps.hpssplhchhat.s.p...                                         cov    pid  321          .         .         :         .         .         .         .         4 400  1 sp|B2ZDY2|L_WWAVU/12219      100.0% 100.0%      SSNYTPTND--------KEISRHKKLLSDCNKIKGLKVLNTRRHTLLCLDVIVLNSLIEMIKLK------INSSQFLINN      2 sp|Q6UY70|L_GTOVV/12198       97.6%  43.1%      QTSLENNFD-EPPLPTTQIEKVYLSLLSACNKIKGKKVFNTRRNTLLFLDLIILNFVAHVYKTQPSEMETLKKAGLIIGE      3 sp|Q6XQI4|L_JUNIN/12210       98.1%  43.2%      EVNMKSQSD-ENSISIDAVERCYLQALSVCNKVKGLKVFNTRRNTLLFLDLVLLNLLCDLFKKHDDAIVRLRNAGIVVGQ      4 sp|Q6IUF8|L_MACHU/12209       98.0%  43.5%      RTDGAATVDQQFSTTHTNIEKHYLSVLSLCNKIKGLKVFNTRRNTLLFLDLIMVNLMVDISESCQDAIESLRKSGLIVGQ      5 sp|B2C4J3|L_CHAVB/12208       98.0%  42.4%      DHSKEEMKD-DEVGGKQPVCISYSSLLSICNKIKGSKIFNTRRNTLLFLDLIMLNFIVDEMIQDDSVVDSLRGAGFIIGQ      6 sp|Q6UY61|L_SABVB/12212       98.1%  42.0%      DVLTRGLNL-DGTKTLHSSVQSYLNVLSMCNKIKGSKIFNTRRNTLLFLDLIMLNFVVDEMVKDSTVIRNLKNAGLIVGQ      7 tr|C5ILC4|C5ILC4_9VIRU/12234  95.9%  33.1%      -EGHPEQDKAEKVGNDRHYHRKSCGMISMLNKSKSIKLMNTRRKLLLGLDVVRLSHLIS---LKNEFPRGDDDESVWCGQ      8 sp|O09705|L_LASSJ/12218       96.1%  33.7%      -N--------EKRGNQTIGECQVQCWRSFLNKVKSLRILNTRRKLLLIFDALILLA--S---KHD--LMKQKCLKGWLGS      9 sp|P14240|L_LYCVA/12210       95.7%  33.3%      ----------HERGSETS--TEYERLLSMLNKVKSLKLLNTRRRQLLNLDVLCLSSLIK---QSK--FKGLKNDKHWVGC        consensus/100%                                  ........................thhS.hNK.Ku.+lhNTRRp.LL.hDhlhl....p....p.......pt.thhhsp        consensus/90%                                   ........................thhS.hNK.Ku.+lhNTRRp.LL.hDhlhl....p....p.......pt.thhhsp        consensus/80%                                   ..........p..hs.p...pph.phlShhNKlKu.KlhNTRRphLLhLDllhLs.hhp....p.....t.cptthhlGp        consensus/70%                                   ...h......c..hs.pt..ppa.phLShhNKlKuhKlhNTRRppLLhLDllhLshlhc...ppp..h.tl+ssthhlGp                                         cov    pid  401          .         .         .         .         :         .         .         . 480  1 sp|B2ZDY2|L_WWAVU/12219      100.0% 100.0%      HFKSVNDRLLSVDLIINKLEKKLLKQPDWLGNVKGKLSK---------------------------RIKPYNLDYVITWL      2 sp|Q6UY70|L_GTOVV/12198       97.6%  43.1%      MLLLPNDRVLDILVARRLLLKKVESCCNWLDRCRHLL-R---------------------------KEEPVLWDCVSEFT      3 sp|Q6XQI4|L_JUNIN/12210       98.1%  43.2%      MLMLVNDRLLDILEAIKLIRKKLMTSPKWVQMCSRTL-K---------------------------NSHQDLWSQLEKLI      4 sp|Q6IUF8|L_MACHU/12209       98.0%  43.5%      MVMLVNDRVLDILEAIKLIRKKIGTNPNWVKNCSKIL-E---------------------------RSHPEIWLQLNTLI      5 sp|B2C4J3|L_CHAVB/12208       98.0%  42.4%      MVVLVNDRALDILAAMKLIRHKLGNSKDWLSVCGKVL-K---------------------------RYDEEMWKEVKTYI      6 sp|Q6UY61|L_SABVB/12212       98.1%  42.0%      MILLVNDRVLDILTANKLIRQKLTTNEKWLSICSSVL-K---------------------------RYDLELWEKLCYLI      7 tr|C5ILC4|C5ILC4_9VIRU/12234  95.9%  33.1%      SFTTVSDRLVSVMSTQRDLKKHLFRLSRMPKNKSKG-KTKALNEIFLDYRRLILNKVFNSLNAHGLTAHSYGLEAS-DFE      8 sp|O09705|L_LASSJ/12218       96.1%  33.7%      CFLRVKDRLVSLEATKRDLEKWGERGNRLRSRITQSSQCLSKNQILNSIFQKTILKATTALKDVGISVDHYKIDM--EVI      9 sp|P14240|L_LYCVA/12210       95.7%  33.3%      CYSSVNDRLVSFHSTKEEFIRLLRNRKK--SKVFR---KVSFEELFRASISEFIAKIQKCLLVVGLSFEHYGLSEHLEQE        consensus/100%                                  .h..spDRhlsh..h.p.h.phh.p..p..t.h.t...p...........................p.c...h.......        consensus/90%                                   .h..spDRhlsh..h.p.h.phh.p..p..t.h.t...p...........................p.c...h.......        consensus/80%                                   hhh.VsDRllsl..s.c.l.+hl.pt.ph.p.htp...p...........................p.c..hhp...ph.        consensus/70%                                   hhhhVNDRllslhtsh+.lc++lhpp.chhp.spph..c...........................p.c..hhpth.phh                                         cov    pid  481          .         5         .         .         .         .         :         . 560  1 sp|B2ZDY2|L_WWAVU/12219      100.0% 100.0%      KELDYEFWYEFKFEREHSARYEKPTLRYKR--QSERVCYQVEFGTDKIFNEEVFLEYLDALSSLSLGMMNSMKTSSATKL      2 sp|Q6UY70|L_GTOVV/12198       97.6%  43.1%      NVPDFELLL----SLAEELCSEKPVMHYKPPSSLIGDCAHKDLM---SMSDGEFESLFKCLSHISLSLVNSMKTSFSSRL      3 sp|Q6XQI4|L_JUNIN/12210       98.1%  43.2%      KHPDMDSMM----ILAQALVSDRPVMRYTIDRESEKICRHQPFS---SLVEGEQKKLFRILSSISLALVNSMKTSFSSRL      4 sp|Q6IUF8|L_MACHU/12209       98.0%  43.5%      RQPDFNSLI----SIAQYLVSDRPIMRYSVERGSDKICRHKLFQ---EMSSFEQMRLFKTLSSISLSLINSMKTSFSSRL      5 sp|B2C4J3|L_CHAVB/12208       98.0%  42.4%      KEPDFDMLF----QLAKSLVSERPIMRYTVHKDNESRCLHQNSL---NISDQSFKAMLKALSHVSLSLINSMKTSFSSRL      6 sp|Q6UY61|L_SABVB/12212       98.1%  42.0%      RVPDFNELF----QLAKELVSDRPMMRYSVHKAEERQCCHKAME---NFTDDDFKIMLKALSHLSLGLINSMKTSFSSRL      7 tr|C5ILC4|C5ILC4_9VIRU/12234  95.9%  33.1%      NWPSLEDFMSF------KKSGSIPVMNYERDNFR--PQMHLD-----ETSFSEDITDLRMISSLSLSLVNSMKTSSVSKI      8 sp|O09705|L_LASSJ/12218       96.1%  33.7%      CLNSYDLIMDF------DVSGVVPTISYQRTEEE--TFPYVM-----GDVELLGTTDLERLSSLSLALVNSMKTSSTVKL      9 sp|P14240|L_LYCVA/12210       95.7%  33.3%      CHIPFTEFENF------MKIGAHPIMYYTKFEDY--NF--QP-----STEQLKNIQSLRRLSSVCLALTNSMKTSSVARL        consensus/100%                                  p..shp.h..........h...hPhh.Yp............................hchlSplsLuhhNSMKTS.ss+l        consensus/90%                                   p..shp.h..........h...hPhh.Yp............................hchlSplsLuhhNSMKTS.ss+l        consensus/80%                                   p..shp.hh.........h.uthPhhpYph.pt....h.........thsp...ht.hchLSplSLuLhNSMKTS.ss+L        consensus/70%                                   p.ssap.hh........thsup+PhMpYph.ptp...pha.......phsp.p.hp.h+tLSplSLuLlNSMKTS.so+L                                         cov    pid  561          .         .         .         6         .         .         .         . 640  1 sp|B2ZDY2|L_WWAVU/12219      100.0% 100.0%      VINDE--KSFYGTVQCEECYFQDLD--SSYNSILLYQKTGEKSRCYGLMLNDDELSDVYRVGQSFYADPKRFFLPIMSSN      2 sp|Q6UY70|L_GTOVV/12198       97.6%  43.1%      LVNEKDYKRYYGTVRLKECYVQRFFLRVGLYGLLFYQKTGEKSRCYSLYLSDKGN---LVELGSFYSDPKRFFLPIFSEF      3 sp|Q6XQI4|L_JUNIN/12210       98.1%  43.2%      LINEREYSRYFGNVRLRECYVQRFHLIKNTFGLLFYQKTGEKSRCYSIYLSINGV---LEEQGSFYCDPKRFFLPIFSED      4 sp|Q6IUF8|L_MACHU/12209       98.0%  43.5%      LVNEREFSKYFGNVRLRECYAQRFYLAESLVGFLFYQKTGERSRCYSVYLSDNGV---MSEQGSFYCDPKRFFLPVFSDE      5 sp|B2C4J3|L_CHAVB/12208       98.0%  42.4%      LINEKDYSRYYGNVRLKECYVQRFPISSRVTGYLFYQKTGERSRCYSLYISENGE---LSELGSFYCDPKRFFLPVFSED      6 sp|Q6UY61|L_SABVB/12212       98.1%  42.0%      LINERDYSRYFGNVRLRECYIQRFPITNNIIGLLFYQKTGERSRCYSLYIAENGE---LTEIGSFYCDPKRYFVPIFSEA      7 tr|C5ILC4|C5ILC4_9VIRU/12234  95.9%  33.1%      RQNASGKN-RYGRVSCEECFFQEIKSPKGTFT-LIYQKTGESSKCYSINR-DGEH------ICSFYADPKRYFLPIMSSE      8 sp|O09705|L_LASSJ/12218       96.1%  33.7%      RQNEFGPA-RYQVVRCKEAYCQEFSLGNTELQ-LIYQKTGECSKCYAINDNKVGE------VCSFYADPKRYFPAIFSAE      9 sp|P14240|L_LYCVA/12210       95.7%  33.3%      RQNQIGSV-RYQVVECKEVFCQVIKLDSEEYH-LLYQKTGESSRCYSIQG-PDGH------LISFYADPKRFFLPIFSDE        consensus/100%                                  h.Nt.....hat.VphcEsahQ.h....t..t.LhYQKTGEpS+CYul.....t.........SFYsDPKRaF.slhSt.        consensus/90%                                   h.Nt.....hat.VphcEsahQ.h....t..t.LhYQKTGEpS+CYul.....t.........SFYsDPKRaF.slhSt.        consensus/80%                                   h.Np.t.t.hatsVphcEsahQch...pt.ht.LhYQKTGEpS+CYul.h..st........tSFYsDPKRaFlPlhSpt        consensus/70%                                   h.N-pt.s.haGsVch+ECYhQch.l.pshhs.LhYQKTGEpSRCYSl.hscsG.......hsSFYsDPKRaFLPIFSpp                                         cov    pid  641          :         .         .         .         .         7         .         . 720  1 sp|B2ZDY2|L_WWAVU/12219      100.0% 100.0%      VILKTCEEMLSWLDWLT--NEETNMIRTKLFTLVINVLTVPSKRVQIYLQGFRYFIMAFVNEFHFKRLNEKLSVQALTSA      2 sp|Q6UY70|L_GTOVV/12198       97.6%  43.1%      VLLATCAEMLSWLDFDE---KLVDAVTPLLKILVLSILSSPTKRSQTFLQGLRYFIMAYVNQAHHIQLMSKLAVECKSAS      3 sp|Q6XQI4|L_JUNIN/12210       98.1%  43.2%      VLIEMCEEMTSWLDFSH---ELMTMTRPTLRLLVLAVLCSPSKRNQTFLQGLRYFLMAYANQIHHVDLMSKLRVDCMSGS      4 sp|Q6IUF8|L_MACHU/12209       98.0%  43.5%      VLAGMCEEMTSWLDFDT---GLMNDTGPILRLLVLAILCSPSKRNQTFLQGLRYFLMAFANQIHHIDLISKLVVECKSSS      5 sp|B2C4J3|L_CHAVB/12208       98.0%  42.4%      TIVSMCNEMVCWLDFDE---QLVELVKPKLRSLVLLLLCSPSKRNQTFIQGLRYFIMAYANQAHHVDLMSKLEIECKSSS      6 sp|Q6UY61|L_SABVB/12212       98.1%  42.0%      VITSMCEEMINWLNFDS---ELVRIVSTQLKTLMLLLLCSPSKRNQTFLQGLRYFIMAYVNQAHHIDLMSKLAVECKSSS      7 tr|C5ILC4|C5ILC4_9VIRU/12234  95.9%  33.1%      VIKGVIDTMVQWLSDVKELEGCIDEIKILLKIIFMSVLLNPSKRSQKFLQNLRYFIMAFFGLAHHVDLMEKVKEDLITKS      8 sp|O09705|L_LASSJ/12218       96.1%  33.7%      VLQTTISTMISWIEDCNELEGQLNNIRSLTKMILVLILAHPSKRSQKLLQNLRYFVMAYLSDYHHKDLIDKIREELITDV      9 sp|P14240|L_LYCVA/12210       95.7%  33.3%      VLYNMIDIMISWIRSCPDLKDCLTDIEVALRTLLLLMLTNPTKRNQKQVQSVRYLVMAIVSDFSSTSLMDKLREDLITPA        consensus/100%                                  sl.thht.MhpWlp......t.hp.ht..hh.lhh.hL..PoKRsQh.lQshRYhlMAhhs.hp.hpL.pKl..phho.s        consensus/90%                                   sl.thht.MhpWlp......t.hp.ht..hh.lhh.hL..PoKRsQh.lQshRYhlMAhhs.hp.hpL.pKl..phho.s        consensus/80%                                   VlhthhppMhsWlp.sp...t.hp.hpshL+hlhl.lLssPoKRsQphlQslRYFlMAahsphHahpLhpKlt.-hhosu        consensus/70%                                   VlhshhppMlsWLc.sp...thhs.lpshL+hLll.lLssPSKRsQpaLQuLRYFlMAassphHHhcLhsKLt.-hhosu                                         cov    pid  721          .         .         :         .         .         .         .         8 800  1 sp|B2ZDY2|L_WWAVU/12219      100.0% 100.0%      EHHVFVLMDELVVCLLEE--ALEENMAKIFKFVLNLSYLCHFITKETPDRLTDQIKCFEKFLEPKLQFGSAFVNLDSSPS      2 sp|Q6UY70|L_GTOVV/12198       97.6%  43.1%      DVLIQRLSVKIVDMVLSDGSDPDMHMTRKFKFVLNVSYLCHLITKETPDRLTDQIKCFEKFMEPKLEFGSLIVNPSLNGF      3 sp|Q6XQI4|L_JUNIN/12210       98.1%  43.2%      EVLIQRMAVELFQTILSEGEDADLYFARRFKYLLNVSYLCHLVTKETPDRLTDQIKCFEKFIEPKVKFDCVVVNPPLNGS      4 sp|Q6IUF8|L_MACHU/12209       98.0%  43.5%      EVVVQRLAVGLFIRLLGGESDASSFFSRRFKYLLNVSYLCHLITKETPDRLTDQIKCFEKFIEPKVKFGCAVVNPSLNGK      5 sp|B2C4J3|L_CHAVB/12208       98.0%  42.4%      EIQLQRLAVTLFELVLSTGDDKDFGFARRFKFLLNISYLCHFVTKETPDRLTDQIKCFEKFLEPKLNFNSVIVNPSLSGI      6 sp|Q6UY61|L_SABVB/12212       98.1%  42.0%      EIQLQRLCVRLFVSILSGDNEIEYGFTRRFKFLLNISYLCHFITKETPDRLTDQIKCFEKFLEPKLKFNSVIVNPSLNGT      7 tr|C5ILC4|C5ILC4_9VIRU/12234  95.9%  33.1%      EYLIYRCVRKVMILVLGD--SVETMLTSRFKFLLNLSYICHFITKETPDRVTDQIKCFEKFLEPKLKFKSVFVNPKN--D      8 sp|O09705|L_LASSJ/12218       96.1%  33.7%      EFLLYRLIRTLMNLVLSE--DVKSMMTNRFKFILNVSYMCHFITKETPDRLTDQIKCFEKFLEPKVRFGHVSTNPAD--T      9 sp|P14240|L_LYCVA/12210       95.7%  33.3%      EKVVYKLLRFLIKTIFGT--GEKVLLSAKFKFMLNVSYLCHLITKETPDRLTDQIKCFEKFFEPKSQFGFFVNPKEA--I        consensus/100%                                  -h.l.hh...lh..lhtt..t.p..hsthFKahLNlSYhCHhlTKETPDRlTDQIKCFEKFhEPK.pFt.h.ss......        consensus/90%                                   -h.l.hh...lh..lhtt..t.p..hsthFKahLNlSYhCHhlTKETPDRlTDQIKCFEKFhEPK.pFt.h.ss......        consensus/80%                                   Eh.l.+hhhtlh.hlLut..s.c.hhsp+FKalLNlSYlCHhlTKETPDRLTDQIKCFEKFhEPKlpFsphhsN.t....        consensus/70%                                   Ehhl.RLhhpLh.hlLup..shc.hhsp+FKFlLNlSYLCHhITKETPDRLTDQIKCFEKFlEPKlpFssshVNPs....                                         cov    pid  801          .         .         .         .         :         .         .         . 880  1 sp|B2ZDY2|L_WWAVU/12219      100.0% 100.0%      LPKEIEDKFVKDINNLFSKDLH----TNDLESPGLSKEILSLCTSCFNCGMLTVGKVLQN---DPQSPSFSSTALDISSN      2 sp|Q6UY70|L_GTOVV/12198       97.6%  43.1%      LSKEQEDVMIEGVEKFFSKELLT---VEDLKRPGVSRELLSYCVSLFNKGRLRVNGTLGT---DPYRPSFTSTALDLSSN      3 sp|Q6XQI4|L_JUNIN/12210       98.1%  43.2%      LTLEQEDIMIRGLDRFFSKEAKT---SSDTQIPGVSKEILSFCISLFNRGRLKVTGELKS---NPYRPNITSTALDLSSN      4 sp|Q6IUF8|L_MACHU/12209       98.0%  43.5%      LTVDQEDIMINGLKKFFSKSLRD---TEDVQTPGVCKELLNYCVSLFNRGKLKVSGELKN---NPFRPNITSTALDLSSN      5 sp|B2C4J3|L_CHAVB/12208       98.0%  42.4%      LTEAQEEIMTSSVNRFFQKNLTN---ISDVKEPGVSKELISFCVSLFNRGKLRVSGDLKV---DPFRPSFTSTALDLSSN      6 sp|Q6UY61|L_SABVB/12212       98.1%  42.0%      LTESQEHQMISSIDRFFSKELLD---QSDVKEPGVSRELLGYCVSLFNRGKLRVSGDLKV---DPFRPTFTSTALDISSN      7 tr|C5ILC4|C5ILC4_9VIRU/12234  95.9%  33.1%      VTKEESSLLLESGSRFLNKDE------FDVKAPGVSKQMLSLMLSFVNSTSGFTPDELAKIKTNPLKDARCSTALDLSSN      8 sp|O09705|L_LASSJ/12218       96.1%  33.7%      ATEEELDDMVYNAKKFLSKDGCTTIEGPDYKRPGVSKKYLSLLTSSFNNGSLFKEREVKREIKDPLITSGS-AALDLASK      9 sp|P14240|L_LYCVA/12210       95.7%  33.3%      TPEEE-CVFYEQMKRFTSKEI-----DCQHTTPGVNLEAFSLMVSSFNNGTLIFKGEKKLNSLDPMTNSGCATALDLASN        consensus/100%                                  hs.t..p.hh.thpphhpKp........php.PGlshphhshhhS.hNps.hhh.t.ht....sP..sshs.sALDluSp        consensus/90%                                   hs.t..p.hh.thpphhpKp........php.PGlshphhshhhS.hNps.hhh.t.ht....sP..sshs.sALDluSp        consensus/80%                                   hs.pp.p.hhpshp+FhsKph.......DhptPGVs+chluhhhS.FNpGpLhhttplt....sPhpsshsuTALDluSN        consensus/70%                                   loc-ppchhlpshp+FhSK-hh.....pDhppPGVS+EhLShhlShFNpGpLhsss-Lp....sPhpsshoSTALDLSSN                                         cov    pid  881          .         9         .         .         .         .         :         . 960  1 sp|B2ZDY2|L_WWAVU/12219      100.0% 100.0%      KSVVVPKLDEVGEIITHYDYQNLLSSVVVEMAQSFKDKLRYKLDRKSVQYAIYRRLTNMVLTKNLNEKNHINENC----E      2 sp|Q6UY70|L_GTOVV/12198       97.6%  43.1%      KSVVIPKLNEVGEIVSEYDKQKLVSTCITSMAERFKTKGRYNLDPDTIDFLIMRNLTNLLSARKLDSSKK--EEL----S      3 sp|Q6XQI4|L_JUNIN/12210       98.1%  43.2%      KSVVIPKLDELGNILSVYDKEKLVSTCVSTMAERFKTKGRYNLDPDSMDYLILKNLTGLVSTGSRTRTNQ--EEL----S      4 sp|Q6IUF8|L_MACHU/12209       98.0%  43.5%      KSVVIPKLDELGNILSTYDKEKLVSACVSSMAERFKTKGRYNLDPESTDYLILKNLTGLVSAGPKAKSSQ--EEL----S      5 sp|B2C4J3|L_CHAVB/12208       98.0%  42.4%      KSVVVPKLDELGNALSKYDKQMMISSCVTTLTEMFKTKGRYNLDPDSLDFLVLKNLSNLVSVSVSKGQMK--EEL----S      6 sp|Q6UY61|L_SABVB/12212       98.1%  42.0%      KSVVVPKLDELGNIVDKYNKQLMVSSCVTSLVEMFKTKGRYNLDPDSIDFLVLKNLTNLVSANVPQEKSQ--EEL----S      7 tr|C5ILC4|C5ILC4_9VIRU/12234  95.9%  33.1%      KSVVIPKTDDKGNSV-DYDYDKLVGVSLCDIVEKFHNKTKFCLSPEQLEYKILKSISHTMAEENRKEKKKKTTTRDSQTS      8 sp|O09705|L_LASSJ/12218       96.1%  33.7%      KSVVVNKYTDGSRIL-NYDFNKLTALAVSQLTEVFSRKGKYLLNKQDYEYKVQQAMSNLVLGSGQLKSDADGAD------      9 sp|P14240|L_LYCVA/12210       95.7%  33.3%      KSVVVNKHLNGERLL-EYDFNKLLVSAVSQITESFVRKQKYKLSHSDYEYKVSKLVSRLVIGSKGEETGRSEDN------        consensus/100%                                  KSVVlsKh.phtp.l..Yshp.hhs.slsphsp.F.pK.+a.Ls.pphpahl.p.hothh..t...tp.t..tp......        consensus/90%                                   KSVVlsKh.phtp.l..Yshp.hhs.slsphsp.F.pK.+a.Ls.pphpahl.p.hothh..t...tp.t..tp......        consensus/80%                                   KSVVlsKhs-huphl.pYDhphhlusslsphsE.FppKt+YpLs.psh-ahl.+tlothl.st..tpptp..pp......        consensus/70%                                   KSVVlPKhs-hGpll.pYDhppLlussVophsEhF+sKs+YpLs.csh-ahlh+slosLV.sps.pcppp..-ph....p                                         cov    pid  961          .         .         .         0         .         .         .         . 1040 1 sp|B2ZDY2|L_WWAVU/12219      100.0% 100.0%      DFEEFLDE-----------DTCRLISDIESNVLECLSNMATPPVRKV-IKG--QEITQRYEGPDLLGRLWSR---ELMGP      2 sp|Q6UY70|L_GTOVV/12198       97.6%  43.1%      LLYEHLSE-----------DVMKAFEEIKYEVEITLSKMRLSRELEC-G----------HKKPCTLEGVWAPF--NVLKV      3 sp|Q6XQI4|L_JUNIN/12210       98.1%  43.2%      MMYESLTE-----------DQVRAFEGIRNDVQMTLAKMANSEGSKV-ETTKLKSKNLSVDERESLESLWAPF--GVLRE      4 sp|Q6IUF8|L_MACHU/12209       98.0%  43.5%      LMYETLTE-----------EQVESFNEIKYDVQVALAKMADNSVNTR-IKNLGRADNSVKNGNNPLDNLWSPF--GVMKE      5 sp|B2C4J3|L_CHAVB/12208       98.0%  42.4%      LLYDTLSE-----------EQLESFEQIKQDVQLTLSRMKESKCNNV-GLGNSRKVNKHLSKSELLETLWSPY--QVLRA      6 sp|Q6UY61|L_SABVB/12212       98.1%  42.0%      TLYEALTE-----------DQISAFEQVRDEVQLALHKMKSSDAREE-RLQDPKRNEKNASKGKILESLWSPH--QVNRA      7 tr|C5ILC4|C5ILC4_9VIRU/12234  95.9%  33.1%      VSESIDLEESEDSVPLEELLPSESLSKIRCSIDKIFSKYQHKQNPKKVQDTEGISVS---EALRD---LVANH--RDRNL      8 sp|O09705|L_LASSJ/12218       96.1%  33.7%      L-DEILLDGG---------A-SDYFDQLKETVEKIVDQYREPVKLGSGPNGDGQP------SINDLDEIVSNK--FYIRL      9 sp|P14240|L_LYCVA/12210       95.7%  33.3%      L-AEICFDGE---------EETSFFKSLEEKVNTTIARYRRGRRANDKGDGEKLTNT---KGLHHLQLILTGKMAHLRKV        consensus/100%                                  ...p...-..............p.hptlc.pl..hhtpht.....t...............t.p....lhs.......t.        consensus/90%                                   ...p...-..............p.hptlc.pl..hhtpht.....t...............t.p....lhs.......t.        consensus/80%                                   h..-hh.-...........t..p.hpplc.pVphhlsphtps...p....t.........pt.p.Lt.lhush..thhp.        consensus/70%                                   h..EhL.E...........-.hc.Fppl+ppVphslu+htpspt.p....s...t.p...pt.p.Lptlhush..tlh+.                                         cov    pid 1041          :         .         .         .         .         1         .         . 1120 1 sp|B2ZDY2|L_WWAVU/12219      100.0% 100.0%      ILAETSLHEVKDFDPSIFSFETYQELCELIFNSE-LKEQFFLDDVLRFCPLELLVKNLTTKNFIEKDFFECFKYILISAG      2 sp|Q6UY70|L_GTOVV/12198       97.6%  43.1%      IRSETSVHEIRDFDPDLLGEDVYEKLCVAVYDSP-LRPTFFLEKPLDICPLELLLKNLTTKSYEDDEFFDCFKYILIQAG      3 sp|Q6XQI4|L_JUNIN/12210       98.1%  43.2%      IKAEVSMHEVKDFDPDVFRSDVYKELCDAVYLSP-FKLTYFLEAPQDICPLGLLLKNLTTIAYQEDEFFECFKYLLIQGH      4 sp|Q6IUF8|L_MACHU/12209       98.0%  43.5%      IRAEVSLHEVKDFDPDVLPSDVYKELCDAVYKSS-EKCNFFLEEVLDVCPLGLLLKNLTTSSYMEEEYFMCFKYLLIQGH      5 sp|B2C4J3|L_CHAVB/12208       98.0%  42.4%      IKNEVSIHEIKDFDPDIIEHETVKKLCDEVYQSS-NKLEFFLEEPLKSVPLEFLLKNLTTIAYEETDYLECFKYLLIQGG      6 sp|Q6UY61|L_SABVB/12212       98.1%  42.0%      IKNETSIHEIKDFDPDILDSHLVEKLCHEVYNSS-QKSLFFLDEPLKSVPLEMLLINLTTIAYEEEEFFECFKYLLIQGD      7 tr|C5ILC4|C5ILC4_9VIRU/12234  95.9%  33.1%      ILSEVSYHLVEDFEESLFPDSFYEDFCERVGNSPKYSKLVMTETVLESCPIEMFTKNITRKFYEEGDWFKCFKTILLQLN      8 sp|O09705|L_LASSJ/12218       96.1%  33.7%      IKGELSNHMVEEFDHDILPGKFYEEFCNAVYENSRLKQKYFYCGHMSQCPIGELTKAVSTRTYFNHEYFQCFKSILLIMN      9 sp|P14240|L_LYCVA/12210       95.7%  33.3%      ILSEISFHLVEDFDPSCLTNDDMKFICEAVEGSTELSPLYFTSVIKDQCGLDEMAKNLCRKFFSENDWFSCMKMILLQMN        consensus/100%                                  IhsEhS.H.lc-F-.shh..p.hp.hC..l..s...p..hhhp...p.sslt.hhhslsp..a.pt-ah.ChK.lLl.ht        consensus/90%                                   IhsEhS.H.lc-F-.shh..p.hp.hC..l..s...p..hhhp...p.sslt.hhhslsp..a.pt-ah.ChK.lLl.ht        consensus/80%                                   IhsEhShH.lcDFD.slh.tchhcchCptVhtSs..p..aFhpt.hc.sPlt.hhKNloph.a.-p-aFpCFKhlLlpht        consensus/70%                                   IhuEsShH.lcDFDPslhspchhcclCctVapSs..+..aFh-tshc.CPLthLhKNLTThsY.Ep-aFpCFKhlLlQhs                                         cov    pid 1121          .         .         :         .         .         .         .         2 1200 1 sp|B2ZDY2|L_WWAVU/12219      100.0% 100.0%      FDNRVGRYDHRSRSRLGFKDAAYHVKEKSRISLRESNSEAVSKRLDKSFFTNSSLRNLCFYSEESPTFQSTVSSSTGKLK      2 sp|Q6UY70|L_GTOVV/12198       97.6%  43.1%      FDQRLGAYEHKNRSRLGLSEEAFRLKEDVRVSNRQSNSEAIADRLDKSFFTSAALRNLCFYSEESPTEYTCISPNVGNLK      3 sp|Q6XQI4|L_JUNIN/12210       98.1%  43.2%      YDQKLGSYEHRSRSRLGFSSEVLKLKDEVRLSTRESNSEAIADKLDKSYFTNAALRNLCFYSDDSPTEFTSISSNTGNLK      4 sp|Q6IUF8|L_MACHU/12209       98.0%  43.5%      FDQKLGSYEHKSRSRLGFTDETLRLKDEVRLSIRESNSEAIADKLDKSYFTNAALRNLCFYSEDSPTEFTSISSNSGNLK      5 sp|B2C4J3|L_CHAVB/12208       98.0%  42.4%      FDQKLGSYEHKSRSRLGLSSEALRVQEDARVSTRESNAEAIAKKLDKTFFTSAALRNLCFYSEDSPTEFTSVSTNTGNLK      6 sp|Q6UY61|L_SABVB/12212       98.1%  42.0%      FDQKLGTYEHKSRSRLGLSSEALKVQENARVSTRESNAEAIAKKLDRTFFTSAALRNLCFYSEDSPTEFTSVSTNTGNLK      7 tr|C5ILC4|C5ILC4_9VIRU/12234  95.9%  33.1%      VNHYSGRFKHNSRLRLNYKFDYKKLYEDARISERESNSEAMCKLLSFTKCITSTLKNLCFYSDESPTSYNGAGPDTGRLQ      8 sp|O09705|L_LASSJ/12218       96.1%  33.7%      ANTLMGRYTHYKSRNLNFKFDMGKLSDDVRISERESNSEALSKALSLTNCTTAMLKNLCFYSQESPQSYDSVGPDTGRLK      9 sp|P14240|L_LYCVA/12210       95.7%  33.3%      ANAYSGKYRHMQRQGLNFKFDWDKLEEDVRISERESNSESLSKALSLTKCMSAALKNLCFYSEESPTSYTSVGPDSGRLK        consensus/100%                                  hsth.GtapH.pp.tLshp.th.+l.-psRlS.RpSNuEuhschLsho.hhsu.L+NLCFYSp-SPp..sshusssGpLp        consensus/90%                                   hsth.GtapH.pp.tLshp.th.+l.-psRlS.RpSNuEuhschLsho.hhsu.L+NLCFYSp-SPp..sshusssGpLp        consensus/80%                                   hsph.GpYcHhpRppLshp.-hh+lp-csRlS.RESNuEAluctLshohhhsusL+NLCFYS--SPTpasslussoGpLK        consensus/70%                                   hsphhGpYcHpsRpRLshp.-hh+lp--sRlSpRESNSEAluctLshohhTsAuL+NLCFYS--SPTpaoolussoGpLK                                         cov    pid 1201          .         .         .         .         :         .         .         . 1280 1 sp|B2ZDY2|L_WWAVU/12219      100.0% 100.0%      FGLSYKEQVGSNRELYVGDLNTKLTSRLIEDYFESITSESKFSCLNNDSEFEKAILDMKSVVRLSGLAVSLDHSKWGPYM      2 sp|Q6UY70|L_GTOVV/12198       97.6%  43.1%      FGLSYKEQVGSNRELYVGDLNTKMMTRLVEDFTEAVANSMNYTCLNSEKEFERAICDMKMAVNNGDLCCSLDHSKWGPFM      3 sp|Q6XQI4|L_JUNIN/12210       98.1%  43.2%      FGLSYKEQVGSNRELYVGDLNTKLMTRLVEDFSEAVGSSMRYTCLNSEKEFERAICDMKMAVNNGDLSCSYDHSKWGPTM      4 sp|Q6IUF8|L_MACHU/12209       98.0%  43.5%      FGLSYKEQVGSNRELYVGDLNTKLMTRLVEDFSEAVGNSMKYTCLNSEKEFERAICDMKMAVNNGDLSCSYDHSKWGPTM      5 sp|B2C4J3|L_CHAVB/12208       98.0%  42.4%      FGLSYKEQVGSNRELYVGDLNTKLMTRLVEDFSEMITSSMRYSCLNSEKEFERAICDMKMAVNNGDISMSLDHSKWGPHM      6 sp|Q6UY61|L_SABVB/12212       98.1%  42.0%      FGLSYKEQVGSNRELYVGDLNTKLMTRLVEDFSEVVTGSMRFSCLNSEKEFERAICDMKMAVNNGDFSLSMDHSKWGPHM      7 tr|C5ILC4|C5ILC4_9VIRU/12234  95.9%  33.1%      FSLSYKEQVGGNRELYIGDMKTKMYTRFVEDFFESLTGQLNGSCLNDEAQFEEAISEMKQCIREGNLSYNMDHSKWGPTM      8 sp|O09705|L_LASSJ/12218       96.1%  33.7%      FSLSYKEQVGGNRELYIGDLRTKMFTRLIEDYFEAISLQLSGSCLNNEKEFENAILSMKLNVSLAHVSYSMDHSKWGPMM      9 sp|P14240|L_LYCVA/12210       95.7%  33.3%      FALSYKEQVGGNRELYIGDLRTKMFTRLIEDYFESFSSFFSGSCLNNDKEFENAILSMTINVREGFLNYSMDHSKWGPMM        consensus/100%                                  FuLSYKEQVGuNRELYlGDhpTKhhoRhlEDa.E.hs...phoCLNs-tpFEpAI.pMp.slp.u.hshshDHSKWGPhM        consensus/90%                                   FuLSYKEQVGuNRELYlGDhpTKhhoRhlEDa.E.hs...phoCLNs-tpFEpAI.pMp.slp.u.hshshDHSKWGPhM        consensus/80%                                   FuLSYKEQVGuNRELYlGDLpTKhhTRLlEDa.EslssphphoCLNs-pEFEpAIhsMK.sVp.utlshShDHSKWGPhM        consensus/70%                                   FuLSYKEQVGuNRELYlGDLpTKhhTRLlEDa.EulssphphoCLNsEKEFEcAIh-MKhsVppGsluhShDHSKWGPhM                                         cov    pid 1281          .         3         .         .         .         .         :         . 1360 1 sp|B2ZDY2|L_WWAVU/12219      100.0% 100.0%      SPAIFNSLFTSLDLQL-R-DGSLIDKSPIENLLNWHLHKLVEVPYNVIEAYLKGYTKRGLGLMDKMSSTVCEDFIFNWF-      2 sp|Q6UY70|L_GTOVV/12198       97.6%  43.1%      SPALFHAFFGALKFKISK-TGEQVDLGPVLNVLKWHLHKAVEVPISVAEAYCTGMLKRRLGLMSLSCQSVCEEFFHQKLL      3 sp|Q6XQI4|L_JUNIN/12210       98.1%  43.2%      SPALFLSFLYTLELKNPR-DRTKVNLEPVMNILKWHLHKVVEVPINVAEAYCVGKLKRSLGLMGCDCTSVGEEFFHQYLQ      4 sp|Q6IUF8|L_MACHU/12209       98.0%  43.5%      SPALFLALLQMLELRTPV-DRSKIDLDSVKSILKWHLHKVVEVPINVAEAYCIGKLKRSLGLMGCGSTSLSEEFFHQTMQ      5 sp|B2C4J3|L_CHAVB/12208       98.0%  42.4%      SPALFYSFLANLNLTEPK-SRAKLNLGPLLDILKWHLHKVVEVPFNVAQAYCVGKIKRSLGLMECQTSSVTEQFYHNFLQ      6 sp|Q6UY61|L_SABVB/12212       98.1%  42.0%      SPALFFTFLANLNLTEPK-SRTRLNLDPLLNILKWHLHKTVEVPFNVAQAYCIGKLKRSLGLMECQCSSLTEEFYHSYLQ      7 tr|C5ILC4|C5ILC4_9VIRU/12234  95.9%  33.1%      CPLLFLTVLKNIKYIKD----FDKPIEHISTLLSWHVHKYVEVPFNVINAITKSFVKRQLGIQQGVINTKTEEFIFSHL-      8 sp|O09705|L_LASSJ/12218       96.1%  33.7%      CPFLFLTVLQNLIFLSKDLQADIKGRDYLSTLLMWHMHKMVEIPFNVVTAMMKSFIKAQLGLRKKTKQSITEDFFYSNF-      9 sp|P14240|L_LYCVA/12210       95.7%  33.3%      CPFLFLMFLQNLKLGDD--QYVRSGKDHVSTLLTWHMHKLVEVPFPVVNAMMKSYVKSKLKLLRGSETTVTERIFRQYF-        consensus/100%                                  sPhlF..hh..l.h..........sht.l.slL.WHhHKhVElPhsVhpAhhhuhhKttLtl.th..pohsEphhhp.h.        consensus/90%                                   sPhlF..hh..l.h..........sht.l.slL.WHhHKhVElPhsVhpAhhhuhhKttLtl.th..pohsEphhhp.h.        consensus/80%                                   sPhLFhshhtsLphh....ph..hsht.l.slLpWHhHKhVEVPhsVhpAhhhuhlKppLGLhtt.ppolsEcFhaphh.        consensus/70%                                   sPhLFhshLtsLphtps..ptsphshs.l.slLpWHlHKhVEVPhNVspAhhhuhlKRpLGLhpttpsolsE-Faaphh.                                         cov    pid 1361          .         .         .         4         .         .         .         . 1440 1 sp|B2ZDY2|L_WWAVU/12219      100.0% 100.0%      AKGQVPSHISSVLDMGQGILHNTSDYYGLVTEQFIMLCLELCFDVRMTAYTSSDDEIMLSNSHSLKDKSDESLDIQKCGE      2 sp|Q6UY70|L_GTOVV/12198       97.6%  43.1%      LEEGVPSHIMSVLDMGQGILHNSSDLYGLITEQFINYCLDFLFDVIPVSYTSSDDQITTFKLPTMSS-SEDGLDGFDWLE      3 sp|Q6XQI4|L_JUNIN/12210       98.1%  43.2%      SRDQVPSHIMSVLDMGQGILHNTSDLYGLITEQFLCYALDLLYDVIPVTYTSSDDQVSLIKIPCLSD--EKCQDRTELLE      4 sp|Q6IUF8|L_MACHU/12209       98.0%  43.5%      LSGQIPSHIMSVLDMGQGILHNTSDLYGLITEQFLCYALDLLYDVIPVSYTSSDDQITLVKTPSLDI--EGGSDAAEWLE      5 sp|B2C4J3|L_CHAVB/12208       98.0%  42.4%      RENEIPSHIMSVLDMGQGILHNLSDLYALITEQFLNYAIYKLYDVDVLSYTSSDDQISIMKLPAYEH--I-DEDSPDWLE      6 sp|Q6UY61|L_SABVB/12212       98.1%  42.0%      IQDEIPSHIMSVLDMGQGILHNLSDLYALITEQFLNYVIHKLFDIDVTSYTSSDDQISIMKLPLSTK--E-NDEDFDWLE      7 tr|C5ILC4|C5ILC4_9VIRU/12234  95.9%  33.1%      DHGIVPSHISSILDMGQGILHNASDYYGLITEKFINYTLKLLFRGELKSFTSSDDQISLFDKELSAT---LRDDTEELLC      8 sp|O09705|L_LASSJ/12218       96.1%  33.7%      QAGVVPSHISSILDMGQGILHNTSDFYALISERFINYAISCICGGTIDAYTSSDDQISLFDQSLTEL---LQRDPEEFRT      9 sp|P14240|L_LYCVA/12210       95.7%  33.3%      EMGIVPSHISSLIDMGQGILHNASDFYGLLSERFINYCIGVIFGERPEAYTSSDDQITLFDRRLSDL---VVSDPEEVLV        consensus/100%                                  ..t.lPSHI.SllDMGQGILHN.SDhYuLloEpFl.hsl.hhht....saTSSDDpl.h.p....t.......-..chh.        consensus/90%                                   ..t.lPSHI.SllDMGQGILHN.SDhYuLloEpFl.hsl.hhht....saTSSDDpl.h.p....t.......-..chh.        consensus/80%                                   .ts.lPSHI.SlLDMGQGILHNhSDhYuLloEpFlsYslthlash...uYTSSDDQIolhc....p.....t.D..-hhp        consensus/70%                                   .ps.lPSHI.SlLDMGQGILHNsSDhYuLITEpFlsYslphlacs..huYTSSDDQIolhch.h.p.....spDs.-hLp                                         cov    pid 1441          :         .         .         .         .         5         .         . 1520 1 sp|B2ZDY2|L_WWAVU/12219      100.0% 100.0%      LLEFHYYLSSKLNKFVSPKTVAGSFASEFKSRFFIWSQEVPLLTKFVAAALHNVKAKSPHQLAETVDTILDQCVANGVSI      2 sp|Q6UY70|L_GTOVV/12198       97.6%  43.1%      LLCFHDFLSSKFNKFVSPKSVSGTFVAEFKSRFFVMGEETPLLTKFVSAALHNVKCKTPTQLAETIDTICDQCVANGVGI      3 sp|Q6XQI4|L_JUNIN/12210       98.1%  43.2%      MVCFHEFLSSKLNKFISPKSVIGTFVAEFKSRFFVMGEETPLLTKFVSAALHNVKCKTPTQLSETIDTICDQCIANGVST      4 sp|Q6IUF8|L_MACHU/12209       98.0%  43.5%      MICFHEFLSSKLNKFVSPKSVIGTFVAEFKSRFFVMGEETPLLTKFVSAALHNVKCKTPTQLSETIDTICDQCIANGVST      5 sp|B2C4J3|L_CHAVB/12208       98.0%  42.4%      IVCFHEYLSSKLNKFVSPKSVVGNFVAEFKSRFFVMGEETPLLTKFVAAALHNVRCKTPTQLAETVDTICDQCVANGVSV      6 sp|Q6UY61|L_SABVB/12212       98.1%  42.0%      IICFHEYLSSKLNKFVSPKSVVGNFVAEFKSRFFVMGEETPLLTKFVAAALHNVKCKTPTQLAETIDTICDQCVANGVGV      7 tr|C5ILC4|C5ILC4_9VIRU/12234  95.9%  33.1%      FLNYHYYLSSQLNKFVSPKSSLCEFVAEFKSRFFVWGEEVPLLTKFVAASLHNVKCKTPHQLSETVDTIVDQCAANGVPI      8 sp|O09705|L_LASSJ/12218       96.1%  33.7%      LIEFHYYMSDQLNKFVSPKSVIGRFVAEFKSRFFVWGDEVPLLTKFVAAALHNIKCKEPHQLAETIDTIIDQSVANGVPV      9 sp|P14240|L_LYCVA/12210       95.7%  33.3%      LLEFQSHLSGLLNKFISPKSVAGRFAAEFKSRFYVWGEEVPLLTKFVSAALHNVKCKEPHQLCETIDTIADQAIANGVPV        consensus/100%                                  hlpap.ahSs.hNKFlSPKos.spFsuEFKSRFalhupEsPLLTKFVuAuLHNl+sKpPpQLsETlDTIhDQshANGVsh        consensus/90%                                   hlpap.ahSs.hNKFlSPKos.spFsuEFKSRFalhupEsPLLTKFVuAuLHNl+sKpPpQLsETlDTIhDQshANGVsh        consensus/80%                                   hlpFH.aLSupLNKFlSPKSVhGpFsAEFKSRFFVhG-EsPLLTKFVuAALHNVKCKpPpQLuETlDTIhDQslANGVsh        consensus/70%                                   hlpFH.aLSSpLNKFVSPKSVhGpFVAEFKSRFFVhGEEsPLLTKFVuAALHNVKCKoPpQLuETlDTIsDQClANGVsl                                         cov    pid 1521          .         .         :         .         .         .         .         6 1600 1 sp|B2ZDY2|L_WWAVU/12219      100.0% 100.0%      KIIKEISKRTNRLISYSGHPIDPFLCVVETDLKDWVDGSRGYRLQRSIESIIADDKQLSIIRNSCKKLFYKIRSGDIQEE      2 sp|Q6UY70|L_GTOVV/12198       97.6%  43.1%      EIVTKISERVNRLIRYSGYPQTPFLAVEKQDVKDWTDGSRGYRLQRNIEHYLQGSEQLEFVRKCAKKVLLKIKKGQVFEE      3 sp|Q6XQI4|L_JUNIN/12210       98.1%  43.2%      HIVSKISIRVNQLIRYSGYRETPFGAIEEQDVKDWVDGSRGYRLQRKIEAIFSDDKETMFIRNCARKVFNDIKKGKIFEE      4 sp|Q6IUF8|L_MACHU/12209       98.0%  43.5%      KIVARISKRVNQLIRYSGYGDTPFGAIEDQDVKDWVDGSRGYRLQRKIEAIFYDDKETSFIRNCARKVFNDIKRGRIFEE      5 sp|B2C4J3|L_CHAVB/12208       98.0%  42.4%      SIVSKISERVNRLVKYSGFGETPFLSVVKQDVKDWSDGSRGYRLQRNIENSLRDSKILEVMRKGARKVFLGIKNGRIFEE      6 sp|Q6UY61|L_SABVB/12212       98.1%  42.0%      DIVSRISERVNRLISYSGYKETPFLTIVNQDVKDWTDGSRGYRLQRNIENSFGNQELLRLIRRGARKVFLEIKKGHVFEE      7 tr|C5ILC4|C5ILC4_9VIRU/12234  95.9%  33.1%      NICNLLQKRCINLLRYCRFPINPFLLNVNTDIKDWIDGTRGYRIQRQLENLFPDS--TGTVRKLVRRLYNSLKNDEVRED      8 sp|O09705|L_LASSJ/12218       96.1%  33.7%      HLCNLIQKRTLNPLQYARYPIDPFLLNCETDVRDWVDGNRSYRIMRQIEGLIPNA--CSKIRSMLRKLYNRLKTGQLHEE      9 sp|P14240|L_LYCVA/12210       95.7%  33.3%      SLVNSIQRRTLDLLKYANFPLDPFLLNTNTDVKDWLDGSRGYRIQRLIEELCPNE--TKVVRKLVRKLHHKLKNGEFNEE        consensus/100%                                  plht.lp.Rs.p.lpYsta..sPFh...ppDl+DW.DGsRuYRl.R.lEt.h.st..h.hhRp.h++lh.tl+psph.E-        consensus/90%                                   plht.lp.Rs.p.lpYsta..sPFh...ppDl+DW.DGsRuYRl.R.lEt.h.st..h.hhRp.h++lh.tl+psph.E-        consensus/80%                                   plss.IpcRs.pLlpYuta..sPFhh..ppDlKDWhDGoRGYRlQRpIEt.h.sp..hthlRphs+Kla.plKpGpl.EE        consensus/70%                                   pIlspIpcRs.pLlpYusas.sPFLh..ppDVKDWsDGSRGYRlQRpIEshh.ss..hphlRphsRKla.clKpGclhEE                                         cov    pid 1601          .         .         .         .         :         .         .         . 1680 1 sp|B2ZDY2|L_WWAVU/12219      100.0% 100.0%      YLVNALQSSPDDCLRQMLRITEVDDQTIEKLIEIRWLNLRAFGDLRLVLRTKIM-SGTRILDREEVPSLIRSVQSKLSKN      2 sp|Q6UY70|L_GTOVV/12198       97.6%  43.1%      YLVQLIGKDGDDALKGFLSYAGCESDEIKDVLKYRWLNLSANGDLRLVLRTKLM-STRRVLEREQIPTLIKTLQSKLSKN      3 sp|Q6XQI4|L_JUNIN/12210       98.1%  43.2%      NLINLISRGGDEALSGFLQYAGCSEDEIRRTLDYRWVNLASFGDLRLVLRTKLM-TSRRVLEKEEMPTLIKTIQSRLSRN      4 sp|Q6IUF8|L_MACHU/12209       98.0%  43.5%      NLINLIGRGGDEALTGFLQYAGCSEQEVNRVLNYRWVNLSSFGDLRLVLRTKLM-TSRRVLEREEVPTLIKTLQSKLSRN      5 sp|B2C4J3|L_CHAVB/12208       98.0%  42.4%      NLIGLIGRGGDEALRGFLLYAEVDKDEIENALRYRWVNTSTFGDLRLVLRTKIM-SSKRVLERESIPSLVKTLQSRMSKN      6 sp|Q6UY61|L_SABVB/12212       98.1%  42.0%      NLIGLIGRGGDEALRGFLLYAGFAENDIVEALRHKWLNPSTFGDLRLVLRTKIM-SSKRILERESVPSLIKTLQSRMSKN      7 tr|C5ILC4|C5ILC4_9VIRU/12234  95.9%  33.1%      FLAELFSRDPFTVLKSLFKHYDLDEP-Q---LRDCWLNLEAHHPIRMVLRQKVLFPSALQIESVNLPTVIRVLQNKLSHS      8 sp|O09705|L_LASSJ/12218       96.1%  33.7%      FTTNYLSSEHLSSLRNLCELLDVEPP-SESDLEYSWLNLAAHHPLRMVLRQKIIYSGAVNLDDEKIPTIVKTIQNKLSST      9 sp|P14240|L_LYCVA/12210       95.7%  33.3%      FFLDLFNRDKTEAILQLGDLLGLEED-LNQLADVNWLNLNEMFPLRMVLRQKVVYPSVMTFQEERIPSLIKTLQNKLCSK        consensus/100%                                  .hhthhtpt..psl.thh.hhtht.......hp.pWlN.tt.hslRhVLRpKlh.sshh.hpp.phPoll+slQs+hspp        consensus/90%                                   .hhthhtpt..psl.thh.hhtht.......hp.pWlN.tt.hslRhVLRpKlh.sshh.hpp.phPoll+slQs+hspp        consensus/80%                                   .hhthhsps..psLpthh.hhthpp....p.lchpWlNhtshtsLRhVLRpKlh.suth.l-cEplPoll+olQs+hSps        consensus/70%                                   .LlsLlu+sss-uLpshhphhshpcs.lpphLchpWlNLssatsLRhVLRpKlh.oothhL-cEplPoLIKTlQs+LS+s                                         cov    pid 1681          .         7         .         .         .         .         :         . 1760 1 sp|B2ZDY2|L_WWAVU/12219      100.0% 100.0%      FVRGAKKIVTDAINKSAFQSSVCSGFIGVCKSMGSKCVRDGSGGFVYIKSLLSEVVCHHTCETC-KPRFSVYCKS-----      2 sp|Q6UY70|L_GTOVV/12198       97.6%  43.1%      FTKGVKKILAESINKSAFQSSVASGFIGFCKSMGSKCVRDGSGGFMYIREVLNKQRVC-PCEICAQNPGIIFCSD-----      3 sp|Q6XQI4|L_JUNIN/12210       98.1%  43.2%      FTKGVKKILAESINKSAFQSSVASGFIGFCKSMGSKCVRDGKGGFLYIKDIFTRIMPC-LCEICEKKPKVIYCQK-----      4 sp|Q6IUF8|L_MACHU/12209       98.0%  43.5%      FTKGVKKILAESINKSAFQSSVASGFIGFCKSMGSKCVRDGKGGFLYIKEVYSGINVC-ICEICALKPKIIYCND-----      5 sp|B2C4J3|L_CHAVB/12208       98.0%  42.4%      FTKGAKKILAESINKSAFQSSVASGFIGFCKSMGSKCVRDGSGGFIYLKDIYKKITTC-ECKHCSVWRGVVYCEK-----      6 sp|Q6UY61|L_SABVB/12212       98.1%  42.0%      FIKGAKKILAESINKSAFQSSVASGFIGFCKSMGSKCVRDGKGGFMYLKELYNNVNKC-GCCICLEWPGVVYCQD-----      7 tr|C5ILC4|C5ILC4_9VIRU/12234  95.9%  33.1%      FTKSTQKLLSDSVNKSAFQSSVASGFIGLAKTIGSKCVRDADRNCHYIVEILSKLESLPGLKVIMKDDIL---IVELESE      8 sp|O09705|L_LASSJ/12218       96.1%  33.7%      FTRGAQKLLSEAINKSAFQSSIASGFVGLCRTLGSKCVRGPNKENLYIKSIQSLISDVKGIKLLTNSNGIQYWQVPLELR      9 sp|P14240|L_LYCVA/12210       95.7%  33.3%      FTRGAQKLLSEAINKSAFQSCISSGFIGLCKTLGSRCVRNKNRENLYIKKLLEDLTTDDHVTRVCNRDGITLYICDKQSH        consensus/100%                                  Fh+uspKlls-ulNKSAFQSslsSGFlGhs+ohGS+CVRs.ptt.hYlhpl.p.......hphh....h...........        consensus/90%                                   Fh+uspKlls-ulNKSAFQSslsSGFlGhs+ohGS+CVRs.ptt.hYlhpl.p.......hphh....h...........        consensus/80%                                   Fs+GspKlLu-uINKSAFQSSluSGFIGhCKohGSKCVRssptt.hYl+plhptl.sh..hphh....hl.hh.......        consensus/70%                                   FT+GspKlLuEuINKSAFQSSVASGFIGhCKohGSKCVRDuptshhYIKclhsplpss.hschs.p.stlhahps.....                                         cov    pid 1761          .         .         .         8         .         .         .         . 1840 1 sp|B2ZDY2|L_WWAVU/12219      100.0% 100.0%      ----------ALERISKYSRSLLWDYFSLVFTNACELGNWVFSCVETPK-----KIPSVVNPNFFWCVKPGSHTELEDKV      2 sp|Q6UY70|L_GTOVV/12198       97.6%  43.1%      ----------ALTLIPEFSRSILWDYFSLVLTNACELGEWVFSSVQPPK-----VPILLNNPNLFWAVKPRGTRLIEDQL      3 sp|Q6XQI4|L_JUNIN/12210       98.1%  43.2%      ----------SLQEVNQFSKPILWDYFSLVLTNACELGEWVFSAVKSPQ-----APLVLCNKNFFWAVKPKAVRQIEDQL      4 sp|Q6IUF8|L_MACHU/12209       98.0%  43.5%      ----------SLNKVSQFSKPILWDYFSLVLTNACELGEWVFSTVKEPQ-----KPLVLNNQNFFWAVKPKVVRQIEDQL      5 sp|B2C4J3|L_CHAVB/12208       98.0%  42.4%      ----------SVEKIFQFTRSIMWDYFTLVLTNACELGEWVFSSVKLPT-----KATILDNPNLFWAIKPRTHKHIEDRL      6 sp|Q6UY61|L_SABVB/12212       98.1%  42.0%      ----------SLAKISQFARSILWDYFTLVLTNACEIGEWVFSDVKSPS-----APPILSNPNLFWAVKPKIQKHIEDRL      7 tr|C5ILC4|C5ILC4_9VIRU/12234  95.9%  33.1%      DGSIQSDWPPECEWLRPMIRPILIDYLCIVLSNCLELGTWVLGDPEPPQ----RGPFLGRSSHDYVAVFPVKSAVLEDKV      8 sp|O09705|L_LASSJ/12218       96.1%  33.7%      N-------GSGGESVVSYFRPLLWDYMCISLSTAIELGAWVLGEPKTVK----VFDFFKHNPCDYFPLKPTASKLLEDRV      9 sp|P14240|L_LYCVA/12210       95.7%  33.3%      --------PEAHRDHICLLRPLLWDYICISLSNSFELGVWVLAEPTKGKNNSENLTLKHLNPCDYVARKPESSRLLEDKV        consensus/100%                                  ..........tht.h..h.+slhhDYhslshosshElG.WVhutsp.sp.....h...h.s.p.ahshhP...t.lEDpl        consensus/90%                                   ..........tht.h..h.+slhhDYhslshosshElG.WVhutsp.sp.....h...h.s.p.ahshhP...t.lEDpl        consensus/80%                                   ..........uhp.l.ph.+slLWDYhslsLoNuhELGtWVhupsp.sp.....hs.hh.N.s.ahslKPt..p.lEDpl        consensus/70%                                   ..........uhppl.pa.RslLWDYhslVLoNAhELGpWVhussc.Pp.....hshhhpNss.aaAlKPpsp+.lEDpl                                         cov    pid 1841          :         .         .         .         .         9         .         . 1920 1 sp|B2ZDY2|L_WWAVU/12219      100.0% 100.0%      NMNHVLYSIKRNFPDLFDEHIAPFLSDLSSLKISWVQRIRFLDLCVAMDMSSECLGVISHIMRRKREESYIVKQNELSLA      2 sp|Q6UY70|L_GTOVV/12198       97.6%  43.1%      GLGHVLQSVRRSYPKVFEEHLVPFMNDLQVSRTTDFTRLRYLDVCVALDMMNENLGIVSHLLKAKDNSIYIVKQSECAVA      3 sp|Q6XQI4|L_JUNIN/12210       98.1%  43.2%      GMNHVLHSIRRNYPKLFEEHLAPFMNDLQVNRSLDSGRLKFLDVCVALDMMNENLGIISHLLKVRDNNVYIVKQSDCASA      4 sp|Q6IUF8|L_MACHU/12209       98.0%  43.5%      GMNHVLQSIRRNYPVLFDEHLAPFMNDLQVSRTMDSGRLKFLDVCIALDMMNENLGIISHLLKTRDNSVYIVKQSDCALA      5 sp|B2C4J3|L_CHAVB/12208       98.0%  42.4%      GLNHILHSIKKNYPQLFEEHLAPFMSDLQSNQMINPSKIKFLDICVALDMVNENLGIIGHLLRGRNNTIYIVKQSECAGA      6 sp|Q6UY61|L_SABVB/12212       98.1%  42.0%      SLNHILHSIKRNYPYLFEEHLAPFMSDLQFNQMMNPSHVKFLDVCIALDMMNENLGIIGHLLRGRNHFIYIVKQSECASA      7 tr|C5ILC4|C5ILC4_9VIRU/12234  95.9%  33.1%      GYNHILQSVRRLYPSLFETHLLPFLDDNSLRKSSWAPKIRFLDLCVSLDITCEAISLISHVVKWKRSEQYTVLTLDLSNS      8 sp|O09705|L_LASSJ/12218       96.1%  33.7%      GLNHIIHSLRRLYPSVFEKHILPFMSDLASTKMKWSPRIKFLDLCVALDVNCEALSLVSHIVKWKREEHYIVLSSELRLS      9 sp|P14240|L_LYCVA/12210       95.7%  33.3%      NLNQVIQSVRRLYPKIFEDQLLPFMSDMSSKNMRWSPRIKFLDLCVLIDINSESLSLISHVVKWKRDEHYTVLFSDLANS        consensus/100%                                  shspll.Sl++.aP.lF-pplhPFhsD.t..p.....+l+aLDlCl.hDh.sEslulluHlh+h+pp..YhVh..-ht.u        consensus/90%                                   shspll.Sl++.aP.lF-pplhPFhsD.t..p.....+l+aLDlCl.hDh.sEslulluHlh+h+pp..YhVh..-ht.u        consensus/80%                                   shNHllpSl+R.YP.lF-cHlhPFhsDhp.pp...ss+l+FLDlClulDh.sEsLulluHll+h+ppp.YhVhps-hu.u        consensus/70%                                   uhNHlLpSl+R.YPplFE-HLhPFMsDLpspphh.ss+l+FLDlCVALDh.sEsLulISHll+h+cpphYIVhpS-hu.u                                         cov    pid 1921          .         .         :         .         .         .         .         0 2000 1 sp|B2ZDY2|L_WWAVU/12219      100.0% 100.0%      HMRDSTPLEGGFQLNSLEICRNFLYQIVFESMLHPVLLTTSQFKKYFWYGEVELLPN-DADHDLGQLTQFIMDCKTLNVS      2 sp|Q6UY70|L_GTOVV/12198       97.6%  43.1%      HIRQVEYVNQELGLSPQQICSNFKIQLVFSSMINPLVITTSVLKSFFWFNEVLNLED-ESQIDVGELTDFTILIKKYNLN      3 sp|Q6XQI4|L_JUNIN/12210       98.1%  43.2%      HVRQSEYTNWEVGISPQQVCRNFMVQVVLSSMINPLVMSTSCLKSFFWFNEVLDLED-DSQVDLAELTDFTLSIKNNKVS      4 sp|Q6IUF8|L_MACHU/12209       98.0%  43.5%      HIRQSSYTDWELGLSPQQICTNFKTQLVLSSMVNPLVLSTSCLKSFFWFNEVLELED-DSQIELAELTDFALMVKNQNVS      5 sp|B2C4J3|L_CHAVB/12208       98.0%  42.4%      HVRQADYVDQDLGLSPQQICYNFKVQFLLSSMINPLIVSTSTLRSFFWFNEVLSIEE-DDQIELGELTDFTLSIKTYNLE      6 sp|Q6UY61|L_SABVB/12212       98.1%  42.0%      HIRQSDYVDHELGLSPQQVCYNFKVQFLFSSMIDPLIVSTSTLKTFFWFNEVLSIEE-EDQIDLGELTDFTLFIKTGHLN      7 tr|C5ILC4|C5ILC4_9VIRU/12234  95.9%  33.1%      HNRKFTTMIDSKVISTSDTCINFLKQLFFESLIRPVLLSSRTLGSFTWFPHASMMPKGEGAPSLGPFENFVLKTIHKGQE      8 sp|O09705|L_LASSJ/12218       96.1%  33.7%      HSRTHEPMVEERVVSTSDAVDNFMRQIYFESYVRPFVATTRTLGSFTWFPHKTSVPEGEGLHRMGPFSSFVEKVIHKGVE      9 sp|P14240|L_LYCVA/12210       95.7%  33.3%      HQRSDSSLVDEFVVSTRDVCKNFLKQVYFESFVREFVATTRTLGNFSWFPHKEMMPSEDGAEALGPFQSFVSKVVNKNVE        consensus/100%                                  H.Rp.p.h..th.lss.phs.NFhhQhhhpShlp.hlhoop.htpa.Wasch..h.p.-s..thu.hppFh..hhp.t.p        consensus/90%                                   H.Rp.p.h..th.lss.phs.NFhhQhhhpShlp.hlhoop.htpa.Wasch..h.p.-s..thu.hppFh..hhp.t.p        consensus/80%                                   H.Rp.p.h..phslSspphC.NFhhQhhhpShlpPhlhoTpsLtsFhWFsch..h.p.-st.plu.hpsFs..hhp.tlp        consensus/70%                                   H.Rpsp.hst-hslSspplChNFhhQlhhpShlpPllhoTpsLtoFhWFscs..l.c.-uthcLG.hosFsh.lhphslp                                         cov    pid 2001          .         .         .         .         :         .         .         . 2080 1 sp|B2ZDY2|L_WWAVU/12219      100.0% 100.0%      RCMSLDDLDVGYVHSKITLSDVFINLSSFIHLLDWGNLCDYESFDKIILESGLEQVPIEIGIVVSHVRRSFKFKYDRKTN      2 sp|Q6UY70|L_GTOVV/12198       97.6%  43.1%      RAMMLDDLTMGYVVSTISEPTIHLVSLKRNSNSIVGEQN----SEMLHGEQVEDMYSIVLHIQLEHKRHSTKYHLSRTVV      3 sp|Q6XQI4|L_JUNIN/12210       98.1%  43.2%      RAMFVEDIAMGYVVSSFDNIKVFLESVSVDNISLLPQEDMIDLHTVLRNVACQEAVKLKLIIQVEHTRVSTKFKLRRKMV      4 sp|Q6IUF8|L_MACHU/12209       98.0%  43.5%      RAMFVEDIAMGYVVSNFEGVRISLSNVMVDGVQLPPKEKAPDVGVLFGLK--AENVIVGLVVQIDHVRMSTKFKLRRKMV      5 sp|B2C4J3|L_CHAVB/12208       98.0%  42.4%      RAMTLDDMTMGYVCSTLLDEVVSLESLDSCQDLAALQFKRQDLSDFFRD-LGEDFVKVGLNIQIVHQRRSTKFDISRKVV      6 sp|Q6UY61|L_SABVB/12212       98.1%  42.0%      RAMTADDITMGYVCSNLAEEIITLNSYGSFQEFRSNHPSKNDLSDILKT-LTSESIKLTLDIQIVHMRNSTKYNISRKIV      7 tr|C5ILC4|C5ILC4_9VIRU/12234  95.9%  33.1%      RPMYRDDLSSGFSWIDLKDFKAYISSAHQRISRVSS---ADAGRDCFNL-----DERVVITITHNFIKRNRNGACDSKFS      8 sp|O09705|L_LASSJ/12218       96.1%  33.7%      RPMFKHDLMMGYAWIDFDIEPARFNQNQLIASGLVDPK-FDSLEDFFDAVASLPPGSAKLSQTVRFRVKSQDASFKESFA      9 sp|P14240|L_LYCVA/12210       95.7%  33.3%      RPMFRNDLQFGFGWFSYRMGDVVCNAAMLIRQGLTNPKAFKSLKDLWDYMLNYTKGVLEFSISVDFTHNQNNTDCLRKFS        consensus/100%                                  RsM.hpDh..Gash.ph....h.h.....................hh..........h.h...h.a.h.p.phth.pphs        consensus/90%                                   RsM.hpDh..Gash.ph....h.h.....................hh..........h.h...h.a.h.p.phth.pphs        consensus/80%                                   RsMhhcDlthGash.sht...h.hpt....t..h.s.....t.tshht.......h.l.l.lpl.ah+.spphphpcphs        consensus/70%                                   RsMhh-DlshGYsh.shp...lhlssht..t..hss..th.shp-hhph..s.p.h.ltlsIplpah+pSpchphpRKhs                                         cov    pid 2081          .         1         .         .         .         .         :         . 2160 1 sp|B2ZDY2|L_WWAVU/12219      100.0% 100.0%      YHIKCKIIIRKSELIMSKVNGVDILEIEVSEIECFVSGSQGHHISLDGVGLIPLHPLFSGKELI-DFNKLLADQSIEFKQ      2 sp|Q6UY70|L_GTOVV/12198       97.6%  43.1%      YSYTVECETNITDIEK------EPSLATVKNVVLRASGSIEGHQFLDGVNLVASQPIFTGKKVI-NLSELLADSEITETY      3 sp|Q6XQI4|L_JUNIN/12210       98.1%  43.2%      YSYTIVSSLRVDDVST------PELELNVDAMSQCVSGSEGNHSLLDGALVIASLPLFTGHESF-DLAGLFIDAGYAVTN      4 sp|Q6IUF8|L_MACHU/12209       98.0%  43.5%      YSFSLECTMDVGDIQN------KEVILKVVAVDQSVSGSGGNHMLLDGVPVIASLPLFTGQASF-DLAAMLIESNLAGSN      5 sp|B2C4J3|L_CHAVB/12208       98.0%  42.4%      YTFRILLLINLSEHLR------EEVKIPVQSLSLYASGAGNNHLFLDGVSMIPTLPLFNGSKSV-NLAKVLIEHELATSN      6 sp|Q6UY61|L_SABVB/12212       98.1%  42.0%      YTLKALCALPLEDCFT------KDPVALVESLELFASGVNGGHLQLDGVTMVSVLPLLRGKKAV-NLAQILMDNDLAATN      7 tr|C5ILC4|C5ILC4_9VIRU/12234  95.9%  33.1%      IIHRIHGSVSDA--------SEGFLMVEVLGVECGFSGAVRDEFVRTNILAIARSLSFSKEDMTVFVQRQMIDQHEEPSN      8 sp|O09705|L_LASSJ/12218       96.1%  33.7%      IHLEYTGSMNQQ--------AKY----LVHDVTVMYSGAVSPCVLSDCWRLVLSGPTFKGKSAW-YVDTEIINEFLIDTN      9 sp|P14240|L_LYCVA/12210       95.7%  33.3%      LIFLVRCQLQNP--------GVA----ELLSCSHLFKGEIDRRMLDECLHLLRTDSVFKVNDGVFDIRSEEFEDYMEDPL        consensus/100%                                  h.h.h...h...................l.th...hpG..t.p...psh.hl.....hphpt.h..htt..hpt......        consensus/90%                                   h.h.h...h...................l.th...hpG..t.p...psh.hl.....hphpt.h..htt..hpt......        consensus/80%                                   h.hph.h.hp.t................V.shp.hhSGs.ttc...-sh.hl...shFpspp.h..ltt.hh-p.ht.s.        consensus/70%                                   hphphhsthp.s..........t...h.V.slphhhSGu.ss+hhhDsl.hlss.PlFpGpcsh.slsp.hh-pthtsop                                         cov    pid 2161          .         .         .         2         .         .         .         . 2240 1 sp|B2ZDY2|L_WWAVU/12219      100.0% 100.0%      -VSSVFQKVKLDFKQHVKELRNKFSYKFQGPEQGLSPLHLYRGQIIERDTIVSRLDVPVTSKSVFLALEALDAADHT-PF      2 sp|Q6UY70|L_GTOVV/12198       97.6%  43.1%      KEGDAVGSILLNFGTFYEHIDDRYAYEIVGPECSDSPLVLDGGSILADGKKLSSIKVELTGDVILKALGALESEKEVQSL      3 sp|Q6XQI4|L_JUNIN/12210       98.1%  43.2%      -DDNILSHVKLNFGDFYSELGNKYAYDLIGPNNPGEPLVLKEGIFYRGNERLSTYKVELSGDVIVKAIGALEDIDSVETL      4 sp|Q6IUF8|L_MACHU/12209       98.0%  43.5%      -DNFLMSNVTLDLGGFSPELSDKYSYRLSGPENQEDPLVLKDGAFYVGGERLSTYKVELTGDLVVKALGALEDDEGVVSM      5 sp|B2C4J3|L_CHAVB/12208       98.0%  42.4%      -DFKLLECVIMDFSNFLDELRDKYSYVLVGPEEQENPIVFQNGAFMADNQKLSYMRVEIFGDTIVKALGALETDREIENL      6 sp|Q6UY61|L_SABVB/12212       98.1%  42.0%      -DHNVMESVTLDFTKFHDELGDKFCYSLVGPEDQGNPIVLHNGMFMIDNQKLSYLKVEIFGDTIIKALGALDSPREIGSL      7 tr|C5ILC4|C5ILC4_9VIRU/12234  95.9%  33.1%      Q--DTIMVELIP--GESFSVDDPLAYTRLGPDWVPVGLVLIDGDVKELKETVAQLKVSLDTKDVMIFLAELYPEH-MALI      8 sp|O09705|L_LASSJ/12218       96.1%  33.7%      QLGHVTPVEIVV-DMER-LQFTEYDFVLVGPCTEPTPLVVHRGGLWECGKKLASFTPVIQDQDLEIFVREVGDTS-SDLL      9 sp|P14240|L_LYCVA/12210       95.7%  33.3%      ILGDSLELELLG-SKRILDGIRSIDFERVGPEWEPVPLTVKMGALFEGRNLVQNIIVKLETKDMKVFLAGLEGY---EKI        consensus/100%                                  .....h...hhs.........p.hsa.h.GPp....slhh..G.hh..tphlt.h.s.l.sp.h.hhlttl.s......h        consensus/90%                                   .....h...hhs.........p.hsa.h.GPp....slhh..G.hh..tphlt.h.s.l.sp.h.hhlttl.s......h        consensus/80%                                   ...phh...hls.tt...ph.sphsa.h.GPp...sPlsl.tG.hh.ttphlu.hpV.l.scslhhhlttLts.p....h        consensus/70%                                   ..tphht..hls.sthh.cltscasYphlGP-pt.sPLVlppGthhtssppluphcVplpscslhhhLuuL-s.c.ht.l                                         cov    pid 2241          :         .         .         .         .         3         .         . 2320 1 sp|B2ZDY2|L_WWAVU/12219      100.0% 100.0%      LKSLHTYMKT----RMSKSNPCFIRMTQEDLCLLIESYEVAFANILKSESDWVEFGDFALCFSNSLNCIMIADDGGQFKL      2 sp|Q6UY70|L_GTOVV/12198       97.6%  43.1%      LTGLWPFIRI---------NNLKVKMAQEDFLLMYEMHRESLLKSLEVFSEWCEFVDFSVCYSKSLRDLVISDSSGSLRL      3 sp|Q6XQI4|L_JUNIN/12210       98.1%  43.2%      LCQLWPYLKT---------TSQTILFQQEDFVLVYDLHKEQLVRSLDKFGDWLEFSNFKVAFSRSLNDLLVSDPQGQFRL      4 sp|Q6IUF8|L_MACHU/12209       98.0%  43.5%      LHQLWPYLKA---------TSQVILFQQEDFTIVHDLYKIQLTKSIESFGEWIEFTNFKVAYSKSLKELVISDTQGSFRL      5 sp|B2C4J3|L_CHAVB/12208       98.0%  42.4%      LCNLWPYLKS---------IKKTIDFNQADFEMIYDLHRTALLKSLCQMDSWIEFTSFSVAYSKHLQDLVVSDNLGNLRL      6 sp|Q6UY61|L_SABVB/12212       98.1%  42.0%      LHGLWPYLKA---------TKQIINFDQTDFEMIYDLHRVVLLESIAQFGDWVEFASFKVAFSKHYKDIVVADNLGNLRL      7 tr|C5ILC4|C5ILC4_9VIRU/12234  95.9%  33.1%      KKGIETMLKKSQKHEVDLSRVDLIKCFK----DVG-LTEHDLVDCFSGVIGWVKLSSYTLAYSKHLKDLVIAHPGGGMRL      8 sp|O09705|L_LASSJ/12218       96.1%  33.7%      IGALSDMMIDRLGLRMQWSGVDIVSTLR----AAAPSCEGILSAVLEAVDNWVEFKGYALCYSKSRGKVMVQSSGGKLRL      9 sp|P14240|L_LYCVA/12210       95.7%  33.3%      SDVLGNLFLHRFRTGEHLLGSEISVILQ----EL-CIDRSILLIPLSLLPDWFAFKDCRLCFSKSRSTLMYEIVGGRFRL        consensus/100%                                  .t.l.shhh.............h..h.p.....h....c..h...ht...tWhth.shtlsaSpphtplhht.s.Gth+L        consensus/90%                                   .t.l.shhh.............h..h.p.....h....c..h...ht...tWhth.shtlsaSpphtplhht.s.Gth+L        consensus/80%                                   hptL.shhht.........t.phl.h.p.....hh..hc..Lhtslp.hspWhcFtsatlsaS+phpplhltps.GphRL        consensus/70%                                   lpsLhshh+t.........ssphl.h.Q....hlhphpc..LhpslpthssWlEFssaplsaSKphpclhlucstGphRL                                         cov    pid 2321          .        ] 2339 1 sp|B2ZDY2|L_WWAVU/12219      100.0% 100.0%      KGRKCRSASTNPR-PLEIE      2 sp|Q6UY70|L_GTOVV/12198       97.6%  43.1%      KGITCKPINLSNS-VTEIE      3 sp|Q6XQI4|L_JUNIN/12210       98.1%  43.2%      KGVTCRPLKHKVE-IKDID      4 sp|Q6IUF8|L_MACHU/12209       98.0%  43.5%      KGVMCRPLANTLQ-VEDIE      5 sp|B2C4J3|L_CHAVB/12208       98.0%  42.4%      KGITCRPFRRDQC-IQEIE      6 sp|Q6UY61|L_SABVB/12212       98.1%  42.0%      KGVTCRLFRQQQS-VEDIE      7 tr|C5ILC4|C5ILC4_9VIRU/12234  95.9%  33.1%      GGVCCRLLREGTSTIE-IE      8 sp|O09705|L_LASSJ/12218       96.1%  33.7%      KGRTCEELTRKDECIEDIE      9 sp|P14240|L_LYCVA/12210       95.7%  33.3%      KGRSCDDWLGGSV-AEDID        consensus/100%                                  tGh.Cc.h..t......I-        consensus/90%                                   tGh.Cc.h..t......I-        consensus/80%                                   KGhpCc.httt.p.hp-I-        consensus/70%                                   KGhsC+.hppp.p.lp-IE ``` |

MView 1.63, Copyright © 1997-2018 Nigel P. Brown
