# Supplementary material for: Purple: A Computational Workflow for Strategic Selection of Peptides for Viral Diagnostics Using MS-Based Targeted Proteomics
Source: Viruses. 2019 Jun 8;11(6):536. doi: 10.3390/v11060536 (PMC6630961; doi:10.3390/v11060536)
Supplement: Supplementary file 1 [file viruses-11-00536-s001.zip › dataS4.html]

MView


|  |
| --- |
| ``` Reference sequence (1): sp|B2ZDY1|Z_WWAVU/195 Identities normalised by aligned length. Colored by: consensus/70% ``` |
| ```                                 cov    pid   1 [        .         .         .         .         :         .         .         . 80  1 sp|B2ZDY1|Z_WWAVU/195      100.0% 100.0%     MGLRYSKDVKDRYGD--REP---EGRIPITLNMPQSLYGRYNCKSCWFANKGLLKCSNHYLCLKCLTLMLRRSDYCGICG     2 sp|Q6UY71|Z_GTOVV/195       96.8%  45.8%     MGNSKSKSNPSSSSE---SQKGAPTVTEFRRTAIHSLYGRYNCKCCWFADKNLIKCSDHYLCLRCLNVMLKNSDLCNICW     3 sp|Q6IVU5|Z_JUNIN/194       95.8%  39.6%     MGNCNGASKSN-QPD---SSRVTQPAAEFRRVAHSSLYGRYNCKCCWFADTNLITCNDHYLCLRCHQVMLRNSDLCNICW     4 sp|Q6IUF9|Z_MACHU/194       95.8%  38.5%     MGNCNKPPKRP-PNT---QTSAAQPSAEFRRTALPSLYGRYNCKCCWFADTNLITCNDHYLCLRCHQTMLRNSELCHICW     5 sp|B2C4J2|Z_CHAVB/198      100.0%  44.9%     MGNTKTKDRQYQSNS--SQPTNTSAPVLLRRQAEPSLYGRHNCRCCWFADTNLVNCSNHYLCLKCLNTMLRRSNLCDICG     6 sp|Q6UY62|Z_SABVB/1100     100.0%  44.0%     MGNSKSKSKLSANQYEQQTVNSTKQVAILKRQAEPSLYGRHNCRCCWFANTNLIKCSDHYICLKCLNIMLGKSSFCDICG     7 tr|C5ILC3|C5ILC3_9VIRU/195  95.8%  38.1%     MGQRHSSGSGQPNPKPSDSD-HEARRS--ELHSDASHLGPLNCKSCWKSKKALVKCYDHYLCLNCLSLLMGITPRCPFCY     8 sp|O73557|Z_LASSJ/199       89.5%  37.6%     MGNK--------QAK-APES-KDSPRA--SLIPDATHLGPQFCKSCWFENKGLVECNNHYLCLNCLTLLLSVSNRCPICK     9 sp|P18541|Z_LYCVA/190       89.5%  30.0%     MGQG--------KSREEKGT-NSTNRA--EILPDTTYLGPLSCKSCWQKFDSLVRCHDHYLCRHCLNLLLSVSDRCPLCK       consensus/100%                               MG.t..............t..........ph....ohhG...C+sCW.t.psLlpC.sHYlChpChphhht.o.hC.hCh       consensus/90%                                MG.t..............t..........ph....ohhG...C+sCW.t.psLlpC.sHYlChpChphhht.o.hC.hCh       consensus/80%                                MGpp.........sp...ps..tt..s..ph.s..ohhG.hsC+sCWhtppsLlpCpsHYLCLpChphhLt.SshCslCh       consensus/70%                                MGpp.t.s....psp...ps.tspshs..ch.s.sShhG.hNCKsCWFuspsLlpCssHYLCL+CLslhLp.SshCsICh                                      cov    pid  81          .         1         .] 111 1 sp|B2ZDY1|Z_WWAVU/195      100.0% 100.0%     EVLPKKLVFENSPSAPPYEA-----------     2 sp|Q6UY71|Z_GTOVV/195       96.8%  45.8%     EQLPTCITVPEEPSAPPE-------------     3 sp|Q6IVU5|Z_JUNIN/194       95.8%  39.6%     KPLPTTITVPVEPTAPPP-------------     4 sp|Q6IUF9|Z_MACHU/194       95.8%  38.5%     KPLPTSITVPVEPSAPPP-------------     5 sp|B2C4J2|Z_CHAVB/198      100.0%  44.9%     EELPTTIIVPVEPSAPLPGQ-----------     6 sp|Q6UY62|Z_SABVB/1100     100.0%  44.0%     EELPTSIVVPIEPSAPPPED-----------     7 tr|C5ILC3|C5ILC3_9VIRU/195  95.8%  38.1%     RELPKNLDLAEAPSAPPL-------------     8 sp|O73557|Z_LASSJ/199       89.5%  37.6%     MPLPTKLRPSAAPTAPPTGAADSIRPPPYSP     9 sp|P18541|Z_LYCVA/190       89.5%  30.0%     YPLPTRLKISTAPSSPPPYEE----------       consensus/100%                               ..LPppl....tPouP...............       consensus/90%                                ..LPppl....tPouP...............       consensus/80%                                ..LPpplhhs.tPoAPP..............       consensus/70%                                c.LPTplhls.tPSAPP.............. ``` |

MView 1.63, Copyright © 1997-2018 Nigel P. Brown
